# Good parent-child relationship protects against alcohol use in maltreated adolescent females carrying the *MAOA*-uVNTR susceptibility allele

**DOI:** 10.3389/fpsyt.2024.1375363

**Published:** 2024-07-22

**Authors:** Megha Bendre, David Checknita, Aniruddha Todkar, Cecilia Åslund, Sheilagh Hodgins, Kent W. Nilsson

**Affiliations:** ^1^ Department of Surgical Sciences, Uppsala University, Uppsala, Sweden; ^2^ Centre for Clinical Research, Uppsala University, Västerås, Sweden; ^3^ Department of Clinical Neuroscience, Karolinska Institute, Stockholm, Sweden; ^4^ Department of Public Health and Caring Sciences, Uppsala University, Uppsala, Sweden; ^5^ Centre de Recherche Institut national de psychiatrie légale Philippe-Pinel and Département de Psychiatrie, Université de Montréal, Montréal, QC, Canada; ^6^ School of Health, Care and Social Welfare, Division of Public Health Sciences, Mälardalen University, Västerås, Sweden

**Keywords:** monoamine oxidase A (MAOA) gene, maltreatment, parent-child relationship, alcohol, adolescents, gene-environment interactions (GxE), differential susceptibility, candidate gene

## Abstract

**Introduction:**

Risk-allele carriers of a Monoamine oxidase A (MAOA) gene, short-allele (MAOA-S) in males and long-allele (MAOA-L) in females, in the presence of a negative environment, are associated with alcohol misuse. Whether MAOA-S/L alleles also present susceptibility to a positive environment to mitigate the risk of alcohol misuse is unknown. Thus, we assessed the association of the three-way interaction of MAOA, maltreatment, and positive parent-child relationship with alcohol consumption among adolescents.

**Methods:**

This prospective study included 1416 adolescents (females: 59.88%) aged 16 - 19 years from Sweden, enrolled in the “Survey of Adolescent Life in Västmanland” in 2012. Adolescents self-reported alcohol consumption, maltreatment by a family (FM) or non-family member (NFM), parent-child relationship, and left saliva for MAOA genotyping.

**Results and discussion:**

We observed sex-dependent results. Females carrying MAOA-L with FM or NFM and a good parent-child relationship reported lower alcohol consumption than those with an average or poor parent-child relationship. In males, the interactions were not significant. Results suggest MAOA-L in females, conventionally regarded as a “risk”, is a “plasticity” allele as it is differentially susceptible to negative and positive environments. Results highlight the importance of a good parent-child relationship in mitigating the risk of alcohol misuse in maltreated individuals carrying genetic risk. However, the interactions were not significant after adjusting to several environmental and behavioural covariates, especially parent’s alcohol use, negative parent-child relationship, and nicotine use (smoking and/or snus), suggesting predictor and outcome intersection. Future studies and frameworks for preventive strategies should consider these covariates together with alcohol consumption. More studies with larger sample sizes are needed to replicate the findings.

## Introduction

Alcohol misuse in early adolescence is a risk factor for alcohol use disorders (AUD) in adulthood (1, 2). AUD is a heterogeneous and heritable reward-deficit disorder that transitions from controlled to uncontrolled, compulsive drinking (3, 4). AUD negatively impacts socioeconomic aspects by increasing the burden of disease, injuries, and mortality (5). The risk of early-onset alcohol use is higher among the offspring of alcoholic parents, partly due to heritability and partly due to environment, as alcoholic parents are likely to maltreat or have a poor relationship with their children (6, 7). Furthermore, adolescents misusing alcohol may also indulge in nicotine use, illicit drug use or violent and non-violent delinquencies (8), further elevating the risk for other mental illnesses in adulthood. Therefore, understanding the predictors of the early onset of alcohol use can help develop strategies to prevent early AUD and promote potential positive health outcomes. However, understanding the predictors of early-onset alcohol use can be intricate as the risk for AUD is moderated by the complex interaction between genes and environment (G×E), both negative and positive, thereby contributing to individual variation in progression from initial alcohol use to AUD (9).

The quality of the early environment contributes to risk and resilience for AUD in adulthood. Children may get adverse effects on behaviour in a proximal environment by parents when they are younger and distally by their neighborhood or peers when they grow older (10). Such negative environmental influences may affect their behaviour in adulthood via the effect on gene expression in the brain. Our preclinical studies in rats show the effect of early life stress on high alcohol intake in adulthood through changes in epigenetics and gene expression of genes involved in the reward systems (11–15). Childhood maltreatment (by a parent or any adult) or poor parent-child relationship are risk factors for early alcohol use, binge drinking, and AUD (7, 16). Childhood maltreated females show a 16-25 fold increased risk of AUD than maltreated males (17) and transition faster from initial alcohol use to AUD (18, 19). In Sweden, maltreatment during adolescence is more common than in childhood, with adolescent males and females differing in their maltreatment experience and the perpetrators (20). Adolescent females reporting poor parent-child relationships are more likely to have early-onset of alcohol use than males (7). In contrast to negative environmental influences, a positive environment, such as a good parent-child relationship, might protect adolescents against maltreatment (21) and early onset of substance use, especially in females (7, 22). Furthermore, a good parent-child relationship is protective against self-motivated alcohol use and attenuates peer-induced drinking behaviour in adolescents (23). Based on the evolutionary perspective of human development, Belsky proposed an operational perspective to study G×E called differential susceptibility to environmental influences, which hypothesizes that individuals differ in their susceptibility to environmental influences, with some being more susceptible to both negative and positive developmental experiences (especially those of rearing environments) and environmental exposures than others (24–26). Recently, a study based on the differential susceptibility framework assessed the interplay between the negative and positive environmental exposures and their effect on alcohol-related problems among youths, where they observed that adolescents and young adults carrying a susceptible allele of the SNP rs2290045 in the *VGLUT2* gene exposed to stressful life events reported more alcohol-related problems if they were also exposed to poor parenting, whereas, in contrast, those exposed to good parenting reported lower alcohol-related problems (27). Another study in adolescents supported the differential susceptibility model by showing that impulsive children exposed to coercive parenting style were at higher risk of alcohol use than those not exposed to coercive parenting style (28). Thus, given the aggravating effect of a negative environment and the mitigating effect of a positive environment on alcohol drinking behaviour, it is critical to include both negative and positive environments to elucidate the effect of G×E on alcohol use behaviour (29).

For the genetic aspect of G×E, we are interested in the Monoamine Oxidase A (*MAOA*), an X-chromosome-linked gene implicated in AUD (30). *MAOA* encodes the monoamine oxidase A enzyme that metabolizes monoamine neurotransmitters (31), which are crucial for reward (3, 32) and stress regulation (33). A functional upstream variable number tandem repeat polymorphism in the promoter region (*MAOA*-uVNTR) is known to regulate *MAOA* gene expression in cell lines, where the short repeats (2 and 3 repeats) underexpress, whereas the long repeats (3.5 and 4 repeats) overexpress the *MAOA* gene (34, 35). *MAOA*-uVNTR is widely studied in the context of G×E, predominantly through the ‘diathesis-stress model’ perspective, associating *MAOA*-uVNTR risk alleles with mental health problems in the presence of a stressful environment in a sex-dependent manner. For example, the short-allele (*MAOA*-S) in men or the long-allele (*MAOA*-L) in womenin the presence of negative environmental factors has been associated with antisocial outcomes (36–39), conduct disorder (40), panic disorder (41), aggression (42–44), anxiety (45), mood disorders (46–49), impulsivity (50); schizophrenia (51) or attention-deficit/hyperactivity syndrome (39). Altogether, the above-mentioned studies based on the classical diathesis–stress model suggest that the *MAOA*-S in males and the *MAOA*-L in females are “ risk alleles” that render the carriers susceptible to the effects of the negative environment (52). A neurobiological explanation is that they have a hyperresponsivity of the amygdala during emotional arousal and reduced amygdala-orbitofrontal connectivity resulting in heightened emotional reactivity to a negative environment (53–56).

Alternatively, studying *MAOA*-uVNTR through the ‘differential susceptibility to environmental influences’ perspective would define the conventional *MAOA*-risk alleles as “plasticity alleles” that render the carriers sensitive to the effects of both negative and positive environments resulting in worst or beneficial outcomes, respectively (57). However, differential susceptibility properties of *MAOA*-uVNTR are not yet investigated. Previous *MAOA*×E studies regarding alcohol consumption have either studied negative or positive environments separately but never together (i.e. in interaction with each other), given that one’s life is a blend of negative and positive experiences. The sex-specific *MAOA*-risk alleles in the presence of a neutral or positive environment have been associated with lower alcohol use, whereas in the presence of a negative environment, has been associated with alcohol misuse and AUD (58–62). However, the picture is incomplete since it is unknown whether the *MAOA*-risk allele carriers experiencing a negative environment benefit from a positive environment compared with non-carriers to protect from alcohol misuse.

Hence, the present study aimed to assess whether the quality of a positive parent-child relationship (a positive environmental factor, Epos) moderates the association of interaction of *MAOA* genotype (candidate gene, cG) and familial or non-familial maltreatment (a negative environmental factor, Eneg) with alcohol misuse in adolescent males and females (i.e. cG×Eneg×Epos). Our second aim was to assess the robustness of the three-way interaction to covariates, such as co-existing negative and positive environments and behavioural outcomes, that can be a risk or resilience factor for harmful alcohol consumption patterns in adolescents. The environmental covariates included parental alcohol use (7), being a victim of bullying in school (63, 64), negative-life events (for example, breaking-up of a romantic relationship with a boyfriend or girlfriend, divorce of a parent, death of a family member, etc.) and positive-life events (for examples, achievements in school or competitions, performed well in exams, etc.) (65). Also, we adjusted the three-way interaction to the negative aspects of a parent-child relationship, such as rejection, chaos and coercion; because adolescents, especially those carrying genetic susceptibility to their environment, could be even more sensitive to the effects of the negative parent-child relationship than the non-carriers (66). Next, because alcohol use, nicotine use (snus or cigarette smoking), illicit drug use and delinquent behaviour are related, and they typically co-occur in adolescents (8, 67–71), we adjusted the three-way interaction to these co-occurring behavioural outcomes to isolate the association of the three-way interaction with alcohol use.

We hypothesize that the *MAOA*-risk alleles would display differential susceptibility to the environment; *MAOA*-risk allele carriers maltreated in early life would report higher or lower alcohol consumption depending on poor or good parent-child relationships, respectively, and benefit more from having a good parent-child relationship than the non-carriers.

## Materials and methods

### Participants

The study includes adolescents born in 1997 and 1999 from Sweden, enrolled in a prospective cohort study, “Survey of Adolescent Life in Västmanland (SALVe cohort)”. These adolescents lived in the medium-sized county of Västmanland, which is representative of the Swedish population due to its mixture of urban and rural areas. In 2012 (wave-1), a total of 4,875 adolescents were contacted by regular mail by retrieving information on personal identity numbers and addresses from the Swedish tax agency. We excluded 163 adolescents due to the following reasons: 138 adolescents had language difficulties, five adolescents had mental disabilities or severe illness, and 20 adolescents moved out of Västmanland County. Of the eligible participants, 1405 adolescents declined to participate, and 1429 did not respond to the survey. A total of 1878 adolescents participated in the study at wave-1. Participants completed a 20-page self-report questionnaire on health, family, school and leisure using paper and pencil. After three years, in 2015 (wave-2), we followed up with the adolescents who participated in wave-1 by contacting them through regular mail, of which 1577 adolescents responded to the survey (attrition rate = 16.03%). Finally, the study sample included 1416 adolescents (Males = 568, Females = 848, Caucasians = 98.7%) who had complete data on alcohol use, genetics and negative and positive environmental variables at wave-2. The mean participant age was 14.4 (12 - 16 years) at wave-1 and 17.3 (16 - 19 years) at wave-2. Regional Board for Research Ethics in Uppsala, Sweden, approved the study (Ref: 2012/187). Adolescents and their parents signed a written informed consent before participation. Participants also left saliva samples at wave-1. The authors were blinded to the individual participant’s identity information during and after the data collection.

### Predictor 1, genetic factor (cG): *MAOA-uVNTR* genotype

We extracted genomic DNA from saliva according to the manufacturer’s guidelines using a DNA Self-Collection Kit (Oragene^®^, Ottawa, Canada). A 30-bp long repeat *MAOA*-uVNTR polymorphism was analyzed as previously described elsewhere (72). We identified five variants of the *MAOA*-uVNTR, where a 30-bp sequence was repeated either 2, 3, 3.5, 4 or 5 times. The *MAOA*-uVNTR 2 and 3 repeats were categorized into short (*MAOA*-S) and 3.5, 4, and 5 repeats into long (*MAOA*-L) alleles. The non-risk allele carriers were coded as 0 and the risk allele carriers as 1 (Males, *MAOA*-L= 0, *MAOA*-S = 1; Females, *MAOA*-S allele (*MAOA*-SS genotype) = 0, *MAOA*-L allele (homozygous *MAOA*-LL and heterozygous *MAOA*-SL genotypes) = 1). In females, the *MAOA*-SL and *MAOA*-LL were grouped together to compare results between males and females, an approach similar to Melas et al. (48). Another reason is that the *MAOA*-SL and *MAOA*-LL females showed similar slopes (**Supplementary Figures S1**, **S2**).

### Predictor 2: negative environment (Eneg)

We collected data on maltreatment by a parent/Family Maltreatment (FM) and maltreatment by any adult/non-familial maltreatment (NFM) at wave-1 and wave-2 through an in-house questionnaire (58). The questionnaire for FM included four questions; Q1) Have there been difficult and abusive arguments between your parents? (witnessed psychological abuse between parents), Q2) Has it happened that any of your parents pushed, hit, or used another violence against the other? (witnessed physical abuse between parents), Q3) Have you been mentally ill-treated (mocked, offended) by any of your parents? (psychological abuse by parents), and Q4) Has it ever happened that any of your parents pushed, hit or used other violence against you? (physical abuse by parents). The first two questions refer to witnessing the FM, and the last two questions refer to experiencing the FM. The participants answered in terms of frequency, coded as 0 = Never, 1 = Yes, once in a year, 2 = Yes, a few times per year, 3 = Yes, a few times a month, 4 = Yes, a few times a week, and 5 = Yes, every or almost every day. The total score for FM ranged from 0-20. The questionnaire for NFM included two questions; Q1) Have you been psychologically maltreated (e.g., mocked, offended) by an adult who does not belong to the family? Q2) Have you been physically maltreated (e.g. beaten, kicked) by an adult who does not belong to the family? Participants answered these questions in frequency by choosing one of the following options coded as 0 - 3; 0 = Never, 1 = Yes, one time, 2 = Yes, 2-4 times, and 3 = Yes, more than five times. The total score for NFM ranged from 0-6. Higher FM or NFM scores indicate severe maltreatment. The main interaction analyses included continuous variables, whereas, for the descriptive, in-depth pairwise comparisons and illustration purpose, we used binary coded variables (FM and NFM variables were 0 = not maltreated, and 1 = maltreated). FM and NFM measured at wave-1 were used as a covariate, and wave-2 data as a predictor in the main analyses (**Supplementary Table S1**).

### Predictor 3: Positive environment (Epos)

In wave-2, the Parents as Social Context Questionnaire (PASCQ) was used to measure adolescents’ perception of their relationship with their parents (73, 74). The positive parent-child relationship included three dimensions; warmth, structure, and autonomy support. Each dimension included four statements (**Supplementary Material and Methods**) where the participants rated each statement on a scale of 0-3 (0 = Not at all true, 1= Not very true, 2= sort of true, and 3= Very true; Cronbach’s alpha = 0.744). The summation index of positive dimensions ranged from 0-36 and was used as a moderator in the main analyses, with a higher score indicating a good parent-child relationship. For descriptive, in-depth pairwise comparisons and illustration purposes, we divided the positive parent-child relationship scale into three groups based on Mean ± one standard deviation (SD): poor (Scores ≤ Mean -1SD = 0 to 23), average (Scores > Mean -1SD but ≤ Mean = 24 to 28), and good (Score ≥ Mean +1SD, = 29 to 36). The summation index of the negative dimension of the parent-child relationship (rejection, chaos, and coercion) (**Supplementary Material and Methods**) was used as a covariate.

### Outcome: Alcohol consumption

At wave-1 and -2, participants completed a three-item questionnaire AUDIT-C, reporting the number of standard drinks, frequency of drinking on a typical day, and frequency of drinking six or more drinks per occasion during the past 12 months (75). The response scale for each item was modified for use in adolescents and is described elsewhere (58). The total AUDIT-C score ranged from 0-14; a higher score indicated higher alcohol consumption. For descriptive and in-depth understanding purposes, we categorized males and females into high and low drinkers based on cut-off for risky alcohol use; AUDIT-C score ≥ 8 for males and ≥ 6 for females, as suggested by a previous study (58). In the main three-way interaction analyses, we used AUDIT-C score at wave-2 as the outcome variable and AUDIT-C score at wave-1 as one of the covariates listed in **Supplementary Table S1**.

### Covariates

We included various environmental covariates such as parents’ AUDIT-C score, negative parent-child relationship, positive life events, negative life events, previous and current exposure to bullying in school (wave-1 and -2), adolescents’ previous exposure to family maltreatment (wave-1), adolescents’ previous exposure to non-family maltreatment (wave-1), and various behavioural covariates which included adolescents’ previous alcohol use (AUDIT-C score at wave-1), together with other behavioural factors that typically co-occur with alcohol use due to cross- genetic transmission (30) such as the use of illicit substances (wave-1), adolescents’ previous and current nicotine use (wave-1 and -2), adolescents’ previous and current involvement in violent and non-violent delinquent behaviours (wave-1 and-2). For a description and list of covariates used for each model, see **Supplementary Material and Methods** and **Supplementary Table S1**. In the *post hoc* analyses, we adjusted the three-way interactions to the above-mentioned environmental and behavioural covariates, according to Keller (76). We used two approaches: an “all-in-one” approach and a “single” approach (details in statistical analyses section under posthoc analyses) to adjust the three-way interaction to all the covariates in one model and to identify which covariates affected the most.

### Statistical analyses

All statistical analyses were performed using the IBM SPSS software (version 24). The analyses were performed separately in males and females due to sex-specific genotypes.

Descriptives: Descriptives for continuous variables are expressed as median (Min-Max) because they were not normally distributed, and categorical variables are expressed as n (%). Between-group differences were tested using a Mann-Whitney *U*-test for continuous variables and Pearson’s chi-square (χ2) test for categorical variables. A comparison of AUDIT-C scores between wave-1 and wave-2 was performed using the Wilcoxon signed ranks test (*Z* statistics). Bivariate correlations were assessed using Spearman's correlation test.

Main Analyses: Three-way interactions cG×E_neg_×E_pos_: Associations of the *MAOA*×FM×positive parent-child relationship and *MAOA*×NFM×positive parent-child relationship with alcohol consumption at wave-2 were assessed using regression-based ordinary least square-based moderated moderation model (77) in SPSS PROCESS macro version 4.2 (IBM, Armonk, NY). The moderated moderation analyses were limited to wave-2 because the data on the positive parent-child relationship was collected only at wave-2. The regression coefficients were estimated using the following equation for the moderated moderation model: Y = *i*_1_+*b*_1_X+*b*_2_M+*b*_3_W+*b*_4_XM+*b*_5_XW+*b*_6_MW+*b*_7_XMW+*e_Y_*, where Y= AUDIT-C scores ranging from 0-14, X= FM scores ranging from 0-20 or NFM scores ranging from 0-6, M= *MAOA*-uVNTR with two categories for each sex, W= positive parent-child relationship score ranging from 0 to 36. The models assessed conditional simple effects (*b*_1_, *b*_2_, *b*_3_), two-way interaction effects (*b*_4_, *b_5_*, *b*_6_) and three-way interaction effect (*b*_7_). *b*_1_ estimates the effect of X on Y when both M and W = 0, *b*_2_ estimates the effect of M on Y when both X and W = 0, and *b*_3_ estimates the effect of W on Y when both X and M = 0, *b_4_* estimates the interaction effect of X and M on Y when W = 0, *b_5_* estimates the interaction effect of X and W on Y when M = 0, *b_6_* estimates the interaction effect of M and W on Y when X = 0, and *b*_7_ estimates the interaction effect of X, M and W on Y. Effects are expressed as unstandardized regression coefficient *b*, standard error, *t*- and *p*-values, and confidence intervals. The change in explained variance (Δ*R^2^*) is reported for significant (*p* ≤ 0.05) three-way interactions. We used scatter plots with jitter for a graphical illustration of three-way interaction to understand the direction of effect and slope of the lines. For this, we first divided the males and females on the basis of parent-child relationship score into three categories: poor, average, and good (see details in predictor 3 section), and then plotted the graph to show how the relationship between AUDIT-C scores and FM or NFM scores change for two categories of *MAOA*-uVNTR genotype. For each line of the *MAOA*-uVNTR genotype, we report explained variation (*R* ^2^) and Spearman correlation coefficient (*rs*) values and *p*-value of *rs* and slope of each line for *MAOA*-uVNTR genotype. Significant three-way interaction(s) were further probed by performing pairwise comparisons using the Kruskal-Wallis test with Bonferroni correction (*adjusted p* = 0.05/24 = 0.002) and illustrated graphically using box plots.

*Post-hoc* analyses: We performed *post-hoc* analyses to 1) understand which of the three dimensions included in the positive parent-child relationship index contributed the most by performing moderated moderation models using W= warmth or structure or autonomy support. 2) Assess the robustness of the significant three-way interactions to various co-existing environmental and behavioural factors as they may also impact alcohol consumption by adjusting for 18 environmental and behavioural covariates (**Supplementary Table S1**). We used two approaches to overcome the problem of overfitting, which reduces the generalizability of the models. We call the first approach an “all-in-one approach” and the second one a “single approach”. In the first all-in-one approach, we conducted a principal component analysis (PCA) on 18 covariates to reduce the number of variables, composited new variables based on the components and included these variables in one model for adjustment as suggested by Keller (76). For PCA, we used all the variables in their original forms without any modification. Covariates passed the Kaiser-Meyer-Olkin Measure (KMO) of the Sampling Adequacy test (KMO values closer to 1 are ideal, but at least 0.05 is acceptable) and Bartlett’s Test of Sphericity (KMO = 0.838 and Bartlett’s Test of Sphericity with χ^2^ (153) = 4319.948, *p* = <0.001) indicating good overlap or strong partial correlation between covariates, a prerequisite for PCA. Using Varimax with Kaiser Normalization for the rotation method, we received five principal components with eigenvalues of more than 1, which cumulatively explained 58.46% of the total variance (**Supplementary Table S2**). We then computed composite variables based on these components. To create composite variables, we used the dichotomous form of each covariate to make the computation ordinal and sensible (described in **Supplementary Methods** for each covariate). We combined all the covariates in Component 1 into one variable called “Behavioural problems 1”, all the covariates in Component 2 into one variable called “Behavioural problems 2”, and all the covariates in Components 3 and 4 were combined into one variable called “Other negative environmental factors” and all the covariates in Component 5 into one variable called “Parent’s alcohol use and Positive life events”. We used these four composited variables and adjusted each of the models with *MAOA*×FM×positive parent-child relationship and *MAOA*×NFM×positive parent-child relationship terms by using general linear models in SPSS to customize the model to include all simple effects, all the possible two- and three-way interaction terms of each composite covariate variables with MAOA genotype, negative environment, and positive environment in one model. In the second, single approach, as the name indicates, we adjusted the three-way interactions to each of the single covariates by including simple effects of MAOA genotype, negative environment, positive environment and a covariate together with all the possible two and three-way interaction terms of a covariate with *MAOA* genotype, negative environment and positive environment. This time, we used the original form of the covariates.

## Results

### Sample description

The differences between males and females regarding the main measures, covariates, and family characteristics are highlighted in **Tables 1**, **2**; **Supplementary Table S3**, respectively. *MAOA*-uVNTR genotypes of females were in Hardy-Weinberg equilibrium (HWE) (*χ* ^2 =^ 0.539, *p* = 0.357). For males, HWE is not reported since their genotype and allelic distribution are similar due to hemizygosity. In both the sexes, the proportion of alcohol drinkers (Males: *U* =35564.000, *p* = 0.091; Females: *U* = 35458.500, *p* = 0.136), participants with a history of FM (Males: *U* = 37728.000, *p* = 0.736; Females: *U* = 35766.000, *p* = 0.197), NFM (Males: *U* = 37432.000, *p* = 0.580; Females: *U* = 37453,000, *p* = 0.606) or perception of parent-child relationship (Males: *U* = 36952.000, *p* = 0.438; Females: *U* = 37387,000, *p* = 0.634) did not significantly differ by *MAOA*-genotype. **Table 1** shows that the median AUDIT-C score at wave-2 was higher than the AUDIT-C score at wave-1 by 3 points in both males (*Z* = -15.729, *p* < 0.001) and females (*Z* = -19.463, *p* < 0.001). Although the proportion of risky alcohol consumers at wave-2 was higher in females compared to males, the AUDIT-C score at wave-2 did not differ significantly between males and females (**Table 1**). In the case of a negative environment, more females, compared to males, reported experiencing FM or NFM at wave-2, particularly psychological maltreatment by a parent or a non-family adult (**Table 1**). For in-depth analyses, we divided the sample into high and low drinkers, and the analyses revealed that the FM and NFM at wave-2 were especially higher among the high-alcohol-drinking females compared to the low-alcohol-drinking females (**Supplementary Table S4**). We also found that high-alcohol-drinking females reported more psychological maltreatment by a parent compared to high-alcohol-drinking males (**Supplementary Table S4**). Next, more than half of the sample lived with their biological parents and siblings (**Supplementary Table S3**), and 55.3% of males and 57.9% of females had a good relationship with their parents, with no significant differences in the quality of the parent-child relationship between males and females (**Table 1**). However, differences emerged when high-alcohol-drinking females were compared with low-alcohol-drinking females, where the proportion reporting poor parent-child relationships was larger among high-alcohol-drinking females (**Supplementary Table S4**).

**Table 1:** Sex-wise descriptive statistics of the wave-2 main measures.

Categorical variables		Males (N=568) *n* (%)	Females (N=848) *n* (%)	Males vs Females *χ* ^2^	Males vs Females *p-*value^¤^	
Caucasians		562 (98.9)	835 (98.5)		
Alcohol users, wave-2		350 (61.6)	534 (63.0)	0.265	0.607
Risky alcohol consumers, wave-2^a^		88 (15.5)	214 (25.2)	19.244	**< 0.001**
*Genetic factor*						
*MAOA*-uVNTR	*MAOA*-S/SS	220 (38.7)	103 (12.1)		
	*MAOA*-L/SL-LL	348 (61.3)	745 (87.9)		
*Negative environmental factors*						
FM, wave-2		248 (43.7)	427 (50.4)	6.106	**0.013**
	Witnessed psychological maltreatment between parents	228 (40.1)	383 (45.2)	3.500	0.061
	Witnessed physical maltreatment between parents	18 (3.2)	42 (5.0)	2.682	0.101
	Experienced psychological maltreatment by parents	59 (10.4)	157 (18.5)	17.379	**< 0.001**
	Experienced physical maltreatment by parents	42 (7.4)	73 (8.6)	0.672	0.412
NFM, wave-2^b^		117 (20.6)	221 (26.1)	5.503	**0.019**
	Experienced psychological maltreatment by a non-family adult	107 (18.9)	220 (25.9)	9.564	**0.002**
	Experienced physical maltreatment by a non-family adult	36 (6.4)	37 (4.4)	2.731	0.098
*Positive environmental factor*						
Positive parent-child relationship, wave-2 only	Poor	104 (18.3)	130 (15.3)	2.255	0.324	
	Average	150 (26.4)	227 (26.8)			
	Good	314 (55.3)	491 (57.9)			
Ordinal variables		Males Median (Min-Max)	Females Median (Min-Max)	Males vs Females *U*	Males vs Females *p-*value^¤^	
AUDIT-C scores, wave-2		3 (0–14)**	3 (0–13)**	237804.0	0.679	
FM scores, wave-2		0 (0–12)	1 (0–12)	217090.0	**0.001**	
NFM scores, wave-2^b^		0 (0–6)	0 (0–6)	227757.0	**0.024**	
Positive parent-child relationship scores, wave-2 only		29 (3–36)	29 (5–36)	226830.0	0.063	

AUDIT-C, Alcohol use disorder identification test-consumption; FM, Family maltreatment; MAOA, Monoamine oxidase A gene; Max, Maximum; Min, Minimum.

NFM, maltreatment by a non-family member.

a cut-off for high-risk alcohol consumption; males ≥ 8 and females ≥ 6, according to Nilsson.2011 et al. (58).

b Data on one male participant missing.

^¤^p-values (2-sided asymptotic significance) ≤ 0.05.

^**^Significant difference in comparison with Wave-1 scores (For AUDIT-C wave-1 scores see **Table 2**).

Significant p-values are marked in bold

**Table 2:** Sex-wise descriptive statistics of covariates.

Covariates (Categorical variables)		Males (N=568) *n* (%)	Females (N=848) *n* (%)	Males vs Females *χ* ^2^	Males vs Females *p-*value^¤^
*FM, Wave-1*		157 (27.6)	241 (28.4)	0.06	0.801
	Witnessed psychological maltreatment between parents	129 (22.7)	210 (24.8)	0.647	0.421
	Witnessed physical maltreatment between parents	9 (1.6)	25 (2.9)	2.658	0.103
	Experienced psychological maltreatment by parents	36 (6.3)	76 (9.0)	3.111	0.078
	Experienced physical maltreatment by parents	34 (6.0)	52 (6.1)	0.007	0.932
*NFM, wave-1*		56 (9.9)	91 (10.7)	0.237	0.626
	Experienced psychological maltreatment by a non-family adult	51 (9.0)	85 (10.0)	0.379	0.538
	Experienced physical maltreatment by a non-family adult	16 (2.8)	15 (1.8)	1.791	0.181
*Negative parent-child relationship, wave-2 only^§^*					
	Low	86 (15.1)	141 (16.6)	2.687	0.442
	Average	246 (43.3)	338 (39.9)		
	High	236 (41.5)	369 (43.5)		
*Bullied in school, wave-1*		182 (32.0)	365 (43.0)	17.362	**< 0.001**
*Bullied in school, wave-2*		153 (26.9)	397 (46.8)	56.589	**< 0.001**
*Parents with alcohol use, wave-1 only*		480 (84.5)	726 (85.6)	0.330	0.566
*Behavioural factors*					
Alcohol users, wave-1		59 (10.4)	111 (13.2)	2.131	0.144
*Nicotine users, wave-1*		29 (5.1)	50 (5.9)	0.337	0.562
*Nicotine users, wave-2*		134 (23.6)	174 (20.5)	1.887	0.170
*Illicit drug users, wave-1 only*		4 (0.7)	3 (0.4)	0.885	0.347
*Involved in non-violent delinquent behaviours, wave-1*		284 (50.0)	364 (42.9)	6.861	**0.009**
*Involved in non-violent delinquent behaviours, wave-2*		335 (59.0)	459 (54.1)	3.251	0.071
*Involved in violent delinquent behaviours, wave-1*		208 (36.6)	100 (11.8)	123.190	**< 0.001**
*Involved in violent delinquent behaviours, wave-2*		220 (38.7)	135 (15.9)	94.235	**< 0.001**
Covariates (Ordinal variables)		Males Median (Min-Max)	Females Median (Min-Max)	Males vs Females *U*	Males vs Females *p-*value^¤^
AUDIT-C scores, wave-1		0 (0–11)	0 (0–11)	226450.0	0.145
FM score, wave-1		0 (0–9)	0 (0–13)	228452.0	0.529
NFM score, wave-1		0 (0–5)	0 (0–5)	230393.0	0.657
Negative parent-child relationship score, wave-2		7 (0-30)	7 (0-33)	236598.0	0.574
Number of positive life events, wave-2		6 (0-16)	6 (0-21)	221402.0	0.056
Number of negative life events, wave-2		2 (0-17)	3 (0-20)	181002.0	**< 0.001**
Frequency of bullying in school, wave-1		0 (0-9)	0 (0-9)	204124.0	**< 0.001**
Frequency of bullying in school, wave-2		0 (0-5)	0 (0-12)	190626.0	**< 0.001**
Parent’s AUDIT-C score, wave-1 only		4 (0-12)	4 (0-12)	229463.5	0.552
Frequency of illicit drug use, wave-1 only		2 (1-11)	2 (1-11)	233493.0	0.459
Non-violent delinquency score, wave-1		0 (0-30)	0 (0-30)	217973.5	**0.004**
Non-violent delinquency score, wave-2		1 (0-44)	1 (0-31)	216049.0	**0.001**
Violent delinquency score, wave-1		0 (0-14)	0 (0-14)	179813.5	**< 0.001**
Violent delinquency score, wave-2		0 (0-22)	0 (0-16)	184259.5	**< 0.001**

^§^Categories are based on Mean ± 1SD.

^¤^p-value is the asymptomatic significance (2-sided).

Significant p-values are marked in **bold.**

AUDIT-C, Alcohol use disorder identification test-consumption; FM, Family maltreatment; Max, Maximum; Min, Minimum; NFM, Non-family maltreatment.

More females than males, especially the high-alcohol-drinking females, reported experiencing other negative events in life and frequent bullying in school at wave-1 and -2 (**Table 2**; **Supplementary Table S5**). At the same time, high-alcohol-drinking males were frequent nicotine users compared to high-alcohol-drinking females (**Supplementary Table S5**). Furthermore, males, compared to females, especially the high-drinking males, were more indulged in violent or non-violent delinquent behaviours (**Table 2**; **Supplementary Table S5**).

### Correlations

Bivariate correlations between variables reported at wave-1 and wave-2 are described in **Supplementary Table S6**. Briefly, the AUDIT-C scores at wave-1 and -2 showed a positive correlation. Alcohol consumption at wave-1 and -2 positively correlated with FM and NFM reported at wave-1 and -2. However, alcohol consumption at wave-2 negatively correlated with the parent-child relationship at wave-2, particularly in females. A positive correlation was observed between and within FM and NFM reported at wave-1 and -2. FM and NFM at wave-2 negatively correlated with the positive parent-child relationship at wave-2.

In summary, descriptive analyses indicate that females, compared to males, were relatively more prone to risky alcohol use; possibly, the risk could be driven by negative environmental exposures at home and outside the home. In the main analyses below, we analyzed whether the risk of alcohol use is moderated by the interaction of *MAOA*-uVNTR genotype, negative and positive environment in a sex-dependent manner.

### Three-way interactions

#### Association of MAOA-uVNTR genotype × FM × positive parent-child relationship with alcohol consumption at wave-2 in females

Females: The three-way interaction between MAOA-uVNTR genotype, FM, and the positive environment was associated with alcohol consumption in females (*n* = 848, *b* = -0.043, *t* (840) = -2.169, *p* = 0.030, *CI* = -0.83 to -0.004), and explained 0.5% variance in the alcohol consumption (*F* (1, 840) = 4.703, *ΔR^2^* = 0.005, *p* = 0.030). Details of the model are described in **Table 3**.

**Table 3:** Description of the model showing three-way interaction effect of *MAOA*-uVNTR genotype, FM, and positive parent-child relationship on alcohol consumption scores at wave-2 in females.

Model^$^	*b*		*se*	*t*	*p*	*LLCI*	*ULCI*
Constant		5.154	2.464	2.091	0.037	0.317	9.992
FM	*b_1_*	-0.207	0.451	-0.458	0.647	-1.092	0.679
*MAOA*-uVNTR genotype	*b_2_*	-1.812	2.625	-0.69	0.490	-6.964	3.34
Positive parent-child relationship	*b_3_*	-0.095	0.082	-1.154	0.249	-0.257	0.067
*MAOA*-uVNTR genotype × FM	*b_4_*	1.048	0.504	2.078	0.038	0.058	2.038
FM × Positive parent-child relationship	*b_5_*	0.018	0.018	0.995	0.320	-0.017	0.053
*MAOA*-uVNTR genotype × Positive parent-child relationship	*b_6_*	0.082	0.088	0.936	0.349	-0.09	0.254
***MAOA*-uVNTR genotype × FM ×** **Positive parent-child relationship**	***b_7_***	**-0.043**	**0.02**	**-2.169**	**0.030**	**-0.083**	**-0.004**

^$^Model: R^2^ = 0.052, F (7,840) = 6.613, p < 0.001. The significant three-way interaction effect is marked in bold. b, beta-coefficient; FM, Family maltreatment; LLCI, Lower limit of confidence interval; se, standard error; t, t-statistics; ULCI, Upper limit of confidence interval.

**Figure 1:**
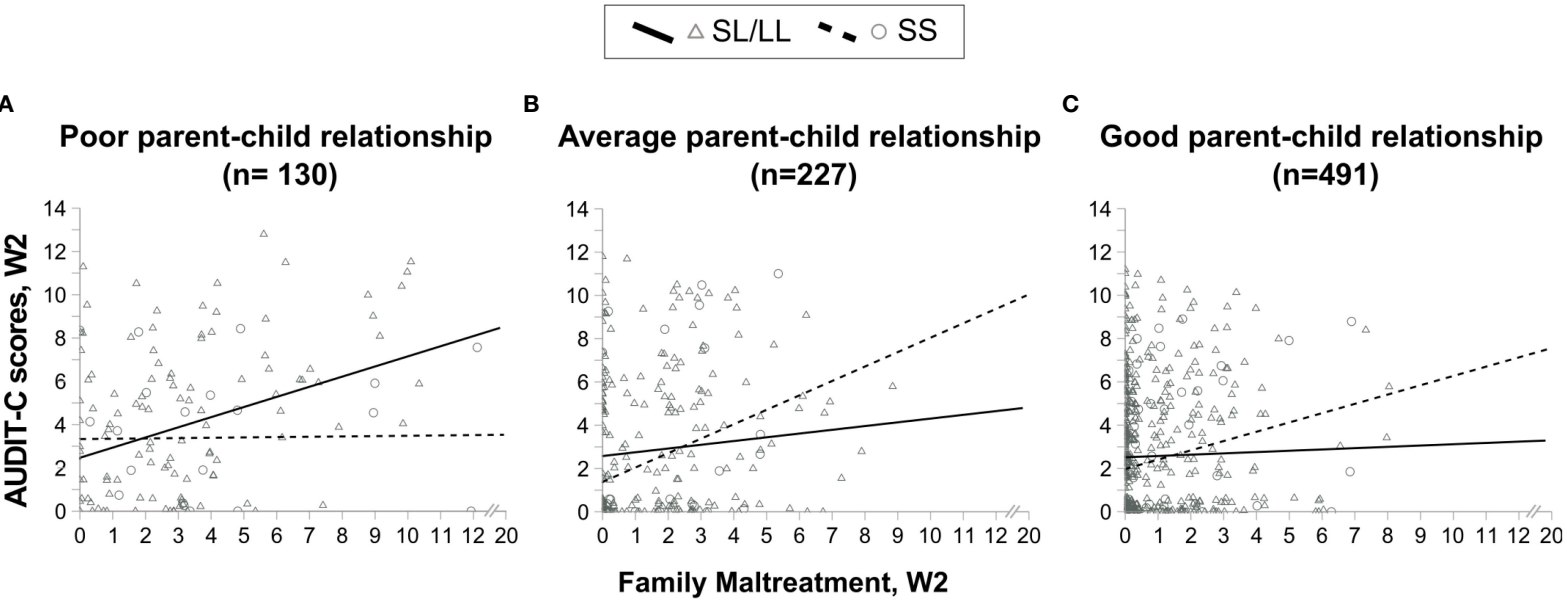
Scatter plots showing the association between alcohol consumption and family maltreatment in *MAOA*-SL/LL and *MAOA*-SS genotype carrying females having **(A)** poor parent-child relationship, **(B)** average parent-child relationship, and **(C)** good parent-child relationship.

A graphical representation of the three-way interaction is shown in **Figure 1**. Among the MAOA-SL/LL carriers who had a poor parent-child relationship, alcohol consumption increased with an increase in FM scores (**Figure 1A**: *n* = 110, *R^2^* = 0.132, *r_s_* = 0.279, *p* = 0.003), whereas those who had average or good parent-child relationship displayed no significant increase in alcohol consumption with an increase in FM scores (**Figure 1B**: *n* = 203, *R^2^* = 0.009, *r_s_* = 0.119, *p* = 0.091, and **Figure 1C**: n = 432, *R^2^* = 0.0008, *r_s_* = 0.034, *p* = 0.487, respectively). However, the MAOA-SS carriers displayed no significant change in alcohol consumption, with the increase in FM scores among those who had either poor (**Figure 1A**: *n* = 20, *R^2^* = 0.00004, *r_s_* = -0.019, *p* = 0.937) or good parent-child relationship (**Figure 1C**: *n* = 59, *R^2^* = 0.066, *r_s_* = 0.224, *p* = 0.088), except a positive trend in those having average parent-child relationship (**Figure 1B**: *n* = 24, *R^2^* = 0.094, *r_s_* = 0.394, *p* = 0.057).

**Figure 2:**
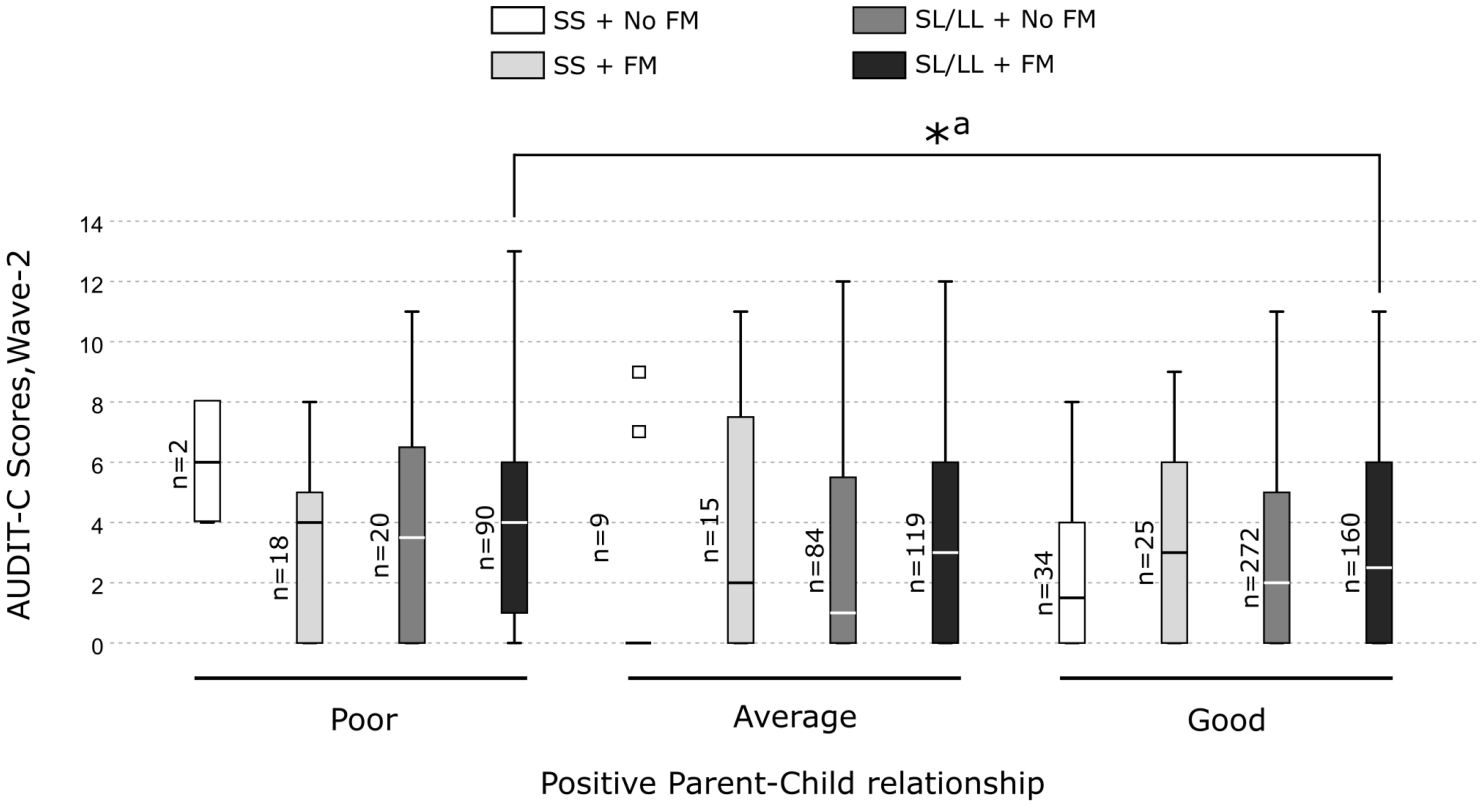
Box plots showing pairwise comparisons to unpack the three-way interaction effect of *MAOA*-uVNTR genotype × family maltreatment × positive parent-child relationship on alcohol consumption in the females. *Significant differences. ^a^ Not significant after Bonferroni correction.

In-depth pairwise comparisons revealed that the MAOA-SL/LL carriers who experienced FM and had a good parent-child relationship displayed significantly lower alcohol consumption compared with those having a poor parent-child relationship (*H* = 86.34, *p* = 0.006) (**Figure 2**). In contrast, among MAOA-SL/LL carriers without FM and MAOA-SS carriers with or without FM, no significant differences in alcohol consumption were observed between those who had a poor, average or good parent-child relationship (**Figure 2**). However, no pairwise comparison was significant after the Bonferroni correction. Additionally, post-hoc analyses revealed that: 1) the three-way interaction containing the warmth dimension was significant and explained the largest variance in alcohol consumption (**Supplementary Table S7A**). A graphical representation of the three-way interaction is shown in **Supplementary Figure S3**. Among the MAOA-SL/LL carriers who had poor warmth, the increase in the AUDIT-C scores for every one unit increase in the FM scores was greater compared to MAOA-SS carriers who had poor warmth (**Supplementary Figure S3A**). In contrast, among MAOA-SL/LL carriers who had good warmth, the increase in the AUDIT-C scores for every one unit increase in the FM scores was lesser compared to MAOA-SS carriers who had good warmth (**Supplementary Figure S3C**). However, among those having average warmth, the increase in the AUDIT-C scores for every one unit increase in the FM scores was similar for both MAOA-SL/LL and MAOA-SS carriers (**Supplementary Figure S3B**). 2) The association of the three-way interaction with alcohol consumption was no longer significant after adjustment for all the covariates using an all-in-one approach (**Supplementary Table S8A**). Specifically, using a single covariate approach, we found that the effect of three-way interaction was sensitive to the effect of all the covariates, except parent’s alcohol use, getting bullied in school at wave-2, and non-violent delinquency at wave-1 (**Supplementary Table S8B**).

Males: The three-way interaction between MAOA-uVNTR genotype, FM, and the positive environment was not associated with alcohol consumption in males (**Supplementary Table S9**, **Supplementary Figures S4**, **S5**).

#### Association of MAOA-uVNTR genotype × NFM × positive Parent-Child relationship with alcohol consumption at wave-2 in females

Females: The three-way interaction between MAOA-uVNTR genotype × NFM × positive parent-child relationship was associated with the alcohol consumption in the females (*n* = 848, *b* = -0.131, *t* (840) = -2.996, *p* = 0.003, *CI* = -0.217 to -0.045) and explained 1% variance in the alcohol consumption (*F* (1, 840) = 8.977, *ΔR^2^* = 0.010, *p* = 0.003). Details of the model are described in **Table 4**.

**Table 4:** Description of the model showing three-way interaction effect of MAOA-uVNTR genotype, NFM, and positive parent-child relationship on alcohol consumption scores at wave-2 in females.

Model^$$^	*b*		*se*	*t*	*p*	*LLCI*	*ULCI*
Constant		6.537	2.032	3.216	0.001	2.548	10.526
NFM	*b_1_*	-1.600	1.019	-1.569	0.117	-3.601	0.401
*MAOA*-uVNTR genotype	*b_2_*	-1.942	2.164	-0.897	0.370	-6.188	2.305
Positive parent-child relationship	*b_3_*	-0.145	0.070	-2.077	0.038	-0.282	-0.008
*MAOA*-uVNTR genotype × NFM	*b_4_*	3.182	1.134	2.807	0.005	0.957	5.407
NFM × Positive parent-child relationship	*b_5_*	0.091	0.040	2.290	0.022	0.013	0.170
*MAOA*-uVNTR genotype × Positive parent-child relationship	*b_6_*	0.089	0.074	1.197	0.232	-0.057	0.235
***MAOA*-uVNTR genotype × NFM × Positive parent-child relationship**	***b_7_***	**-0.131**	**0.044**	**-2.996**	**0.003**	**-0.217**	**-0.045**

^$$^Model: (R^2^ = 0.068, F (7,840) = 8.786, p < 0.001). The significant three-way interaction effect is marked in bold. b, beta-coefficient; LLCI, Lower limit of the confidence interval; NFM, maltreatment by a non-family member; se, standard error; t, t-statistics; ULCI, Upper limit of the confidence interval.

**Figure 3:**
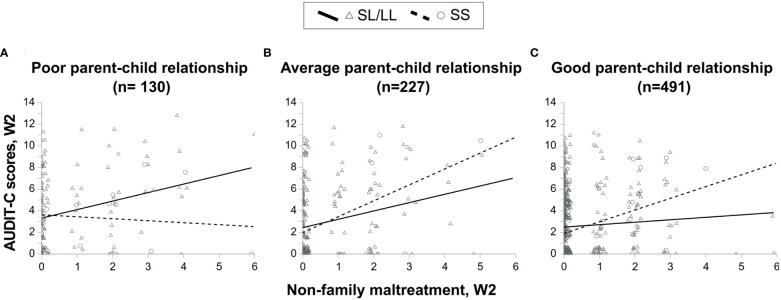
Scatter plots showing the association between alcohol consumption and non-family maltreatment in *MAOA*-SL/LL and *MAOA*-SS genotype-carrying females having **(A)** poor parent-child relationship, **(B)** average parent-child relationship, and **(C)** good parent-child relationship.

A graphical representation of the three-way interaction is shown in **Figure 3**. The MAOA-SL/LL carriers who had a poor parent-child relationship displayed an increase in alcohol consumption with an increase in NFM scores compared to *MAOA*-SS carriers who had a poor parent-child relationship (**Figure 3A**: MAOA-SL/LL carriers: *n* = 110, *R^2^* = 0.086, *r_s_* = 0.228, *p* = 0.017; MAOA-SS carriers: *n* = 20, *R^2^* = 0.011, *r_s_* = -0.066, *p* = 0.781). In contrast, MAOA-SL/LL carriers having an average or a good parent-child relationship displayed less or even lesser increase in alcohol consumption with every one unit an increase in NFM scores, compared to MAOA-SS carriers who had an average or a good parent-child relationship, respectively (**Figure 3B**: MAOA-SL/LL carriers: *n* = 203, *R^2^* = 0.059, *r_s_* = 0.223, *p* = 0.001; MAOA-SS carriers: *n* = 24, *R^2^* = 0.211, *r_s_* = 0.431, *p* = 0.035, and **Figure 3C**: MAOA-SL/LL carriers: *n* = 432, *R^2^* = 0.004, *r_s_* = 0.069, *p* = 0.150; MAOA-SS carriers: *n* = 59, *R^2^* = 0.129, *r_s_* = 0.278, *p* = 0.033, respectively).

**Figure 4:**
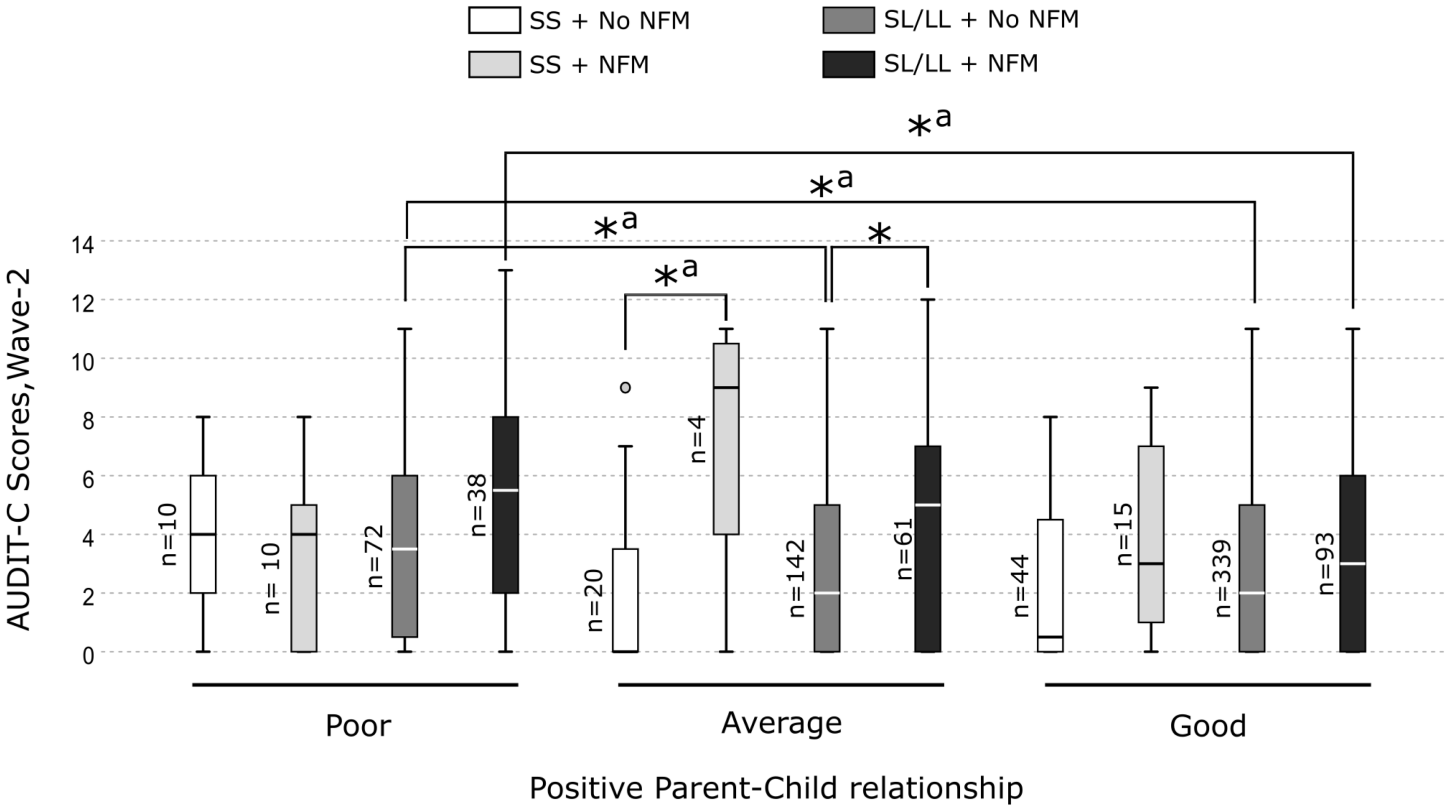
Box plots showing pairwise comparisons to unpack the three-way interaction effect of *MAOA*-uVNTR genotype × non-family maltreatment × positive parent-child relationship on alcohol consumption in the females.*Significant differences. ^a^ Not significant after Bonferroni correction.

Further, the pairwise comparisons revealed that the MAOA-SL/LL carriers who experienced NFM and had a good parent-child relationship displayed significantly lower alcohol consumption than those who had a poor parent-child relationship (*H* = 120.95, *p* = 0.008). Also, the MAOA-SL/LL carriers without NFM and having a poor parent-child relationship reported higher alcohol consumption compared with those with an average or a good parent-child relationship (*H* = 84.37, *p* = 0.014, and *H* = 69.33, *p* = 0.025, respectively) (**Figure 4**). Whereas, among the MAOA-SS carriers with or without NFM, no significant differences in alcohol consumption were observed between those who had a poor, average or good parent-child relationship (**Figure 4**). However, no pairwise comparison was significant after the Bonferroni correction.

Post-hoc analyses revealed that: 1) The three-way interactions containing the warmth and autonomy support dimension were significant, whereas the structure dimension was borderline significant. The warmth dimension explained the largest variance in alcohol consumption (**Supplementary Table S7B**). A graphical representation of the three-way interaction with each dimension is shown in **Supplementary Figure S6** Among the MAOA-SL/LL carriers who had poor warmth, structure or autonomy support, the increase in the AUDIT-C scores for every one unit increase in the NFM scores was greater compared to MAOA-SS carriers who had poor warmth, structure or autonomy support respectively (**Supplementary Figures S6A, D, G**). In contrast, among MAOA-SL/LL carriers who had good warmth, structure or autonomy support, the increase in the AUDIT-C scores for every one unit increase in the NFM scores was lesser compared to MAOA-SS carriers who had an average (**Supplementary Figures S6B, E, H**) or good warmth, or structure or autonomy support, respectively (**Supplementary Figures S6C, F, I**). 2) The association of the three-way interaction between MAOA-uVNTR genotype, NFM, and the positive environment with alcohol consumption was not significant after adjusting to all the covariates using an all-in-one approach (**Supplementary Table S10A**). However, using a single approach, we observed that the association of the three-way interaction with alcohol consumption was robust to the effects of all the environmental and behavioural covariates except for parent’s alcohol use, negative parent-child relationship and adolescents’ current nicotine use (Wave-2) (**Supplementary Table S10B**).

*Males:* The three-way interaction between *MAOA*-uVNTR genotype, NFM, and the positive environment was not associated with alcohol consumption in males (**Supplementary Table S11**, **Supplementary Figures S7**, **S8**).

## Discussion

The present cG×Eneg×Epos study demonstrates for the first time the differential susceptibility of *the MAOA*-uVNTR genotype to negative and positive environments constituting risk or protection against alcohol misuse among Swedish adolescents. We used a differential susceptibility to environmental influences framework. We used separate constructs for FM, NFM (negative environment), and positive-parent child relationship (positive environment) reported at wave-2. Using separate constructs for correlated parent-related variables, FM and positive-parent child relationship, enabled us to isolate the moderating effect of positive-parent child relationship on effect of FM or NFM on alcohol use. However, taking into consideration a possible correlation between various negative environmental factors, in the *post-hoc* analyses, we have adjusted the three-way interactions to the maltreatments reported at wave-1, negative-parent-child relationship and other covariates (**Supplementary Tables S1**, **S2**, **S8A, B**, **S10A, B**), and we have reported both unadjusted and adjusted three-way interactions. In the unadjusted analyses, we observed that the females carrying the *MAOA*-L allele (*MAOA*-SL/LL genotype) with FM or NFM reported lower alcohol use in the presence of a good parent-child relationship (specifically, warmth dimension), whereas, reported higher alcohol use in the presence of a poor parent-child relationship compared to their counterparts carrying *MAOA*-SS genotype (**Figures 1**–**4**). The *MAOA*-L allele carriers benefited from a good parent-child relationship to counteract the negative effects of maltreatment on alcohol use, whereas *MAOA*-SS genotype carriers did not. Thus, the *MAOA*-L allele is sensitive to negative and positive environmental factors, thereby displaying characteristics of a “plasticity” allele rather than a “risk” allele. Thus, *MAOA* plasticity allele carriers in a negative environment may not always be at risk of alcohol misuse and develop AUD in adulthood because the positive environment may mitigate the risk. Hence, promoting a positive parent-child relationship could be a preventive strategy for mitigating the risk of AUD amongst *MAOA* plasticity allele carriers. Among *MAOA*-S allele-carrying females, the opposing effects of a good parent-child relationship on their alcohol consumption need further investigation, although such an unexcepted pattern may exist in some families (78).

The present study’s findings cannot be directly compared with earlier *MAOA*×E studies on alcohol because they have only accounted for the negative environment and have reported higher alcohol consumption among females carrying the *MAOA*-L allele (58, 62) with some discrepancies (61, 79). Nevertheless, further replications of the findings are needed in different populations with varying degrees of negative and positive environmental factors. The findings of the present study underscore the importance of studying a positive environment to help explain inconsistencies in earlier *MAOA*×Eneg studies. Also, the differential susceptibility of the *MAOA*-L allele could explain the lack of association between the *in vivo* transcriptional activity of *MAOA*-alleles (80), otherwise clearly demonstrated in *in-vitro* (34). Additionally, understanding the biological “how” and “why” underlying differential susceptibility of the *MAOA*-L allele may help develop preventive intervention strategies for AUD treatment.

The intermediate phenotype of emotional regulation and epigenetic mechanisms such as DNA methylation might help to explain the observed differential susceptibility of the *MAOA*-L allele. A study in females carrying the *MAOA*-L allele exposed to childhood maltreatment observed heightened emotional reactivity during adolescence that predicted pathological personality in adulthood, suggesting emotional reactivity as a mediator between *MAOA*×Eneg and psychiatric illness (56). A good parent-child relationship, however, facilitates effective emotion regulation among adolescents in high-risk environments (81). Thus, we speculate that the protective effect of a good parent-child relationship (specifically, warmth dimension) in *MAOA*-L allele carriers might have mitigated the heightened emotional reactivity to the negative environment, resulting in lower alcohol consumption. Besides, DNA methylation is implicated in emotion regulation (82, 83), and it might translate environmental cues to genes and influence the development of psychiatric disorders (84). In general, DNA methylation is sensitive to the early environment (85), and alcohol consumption (13, 86), and specifically, *MAOA* methylation and transcription factors regulate the *MAOA* allelic expression in response to environmental changes (87). Hence, the parent-child relationship-induced changes in *MAOA* methylation may have contributed to emotional reactivity and differential susceptibility of plasticity alleles. However, further studies are needed to elucidate such a molecular mechanism.

The cG×Eneg×Epos was associated with alcohol consumption in females but not males (**Supplementary Tables S9**, **S11**, **Supplementary Figures S4**, **S5**, **S7**, **S8**) probably because of a higher proportion of high-alcohol-drinkers in females than males (25.23% vs 15.49%). In the present study, the lower number of male drinkers in the statistical models might have too few cases to detect the association of cG×Eneg×Epos interaction with alcohol consumption. The observed drinking trend, however, aligns with the recent reports among Swedish adolescents, where alcohol drinking is more common among girls than boys (88, 89). Moreover, a higher proportion of girls than boys in Sweden reported alcohol consumption at least once during their lifetime or intoxication during the past 30 days (90). Another reason for associations in females could be because, in our study, the high-alcohol-drinking females reported higher frequencies of psychological maltreatment, bullying in school and negative life events relative to the high-alcohol-drinking males (**Supplementary Table S5**). Presumably, similar to females, the interaction effect on alcohol consumption would be evident in males at a higher severity of maltreatment, as shown by a study concerning antisocial outcomes (91). Also, in males, co-occurring nicotine usage and involvement in delinquent behaviours might have interfered with the associations with alcohol use. Furthermore, in contrast to females, males, irrespective of genotype, did not show sensitivity to the quality of the parent-child relationship; they instead displayed a trend of higher alcohol consumption in the presence of a good parent-child relationship (**Supplementary Figures S4**, **S7**). Females have a stronger perception of stress, are strongly affected by the quality of their relationship with their parents, and prefer to seek emotional support as a coping mechanism relative to males (7, 92, 93). Thus, having a good parent-child relationship (warmth, autonomy support, and structure) might have emotionally supported the females to mitigate stress and alcohol consumption.

Moreover, we adjusted the three-way interactions for predictor- and outcome-intersection problems, which are common in G×E studies. Predictor-intersection means the intersection of co-occurring and re-occurring maltreatment that cumulatively may predict mental health outcome(s) (94). For example, we found a considerable overlap between FM and NFM measured at two-time points; in females who experienced FM at wave-1, 19.9% also experienced NFM, while 75.9% experienced FM and 38.2% experienced NFM at wave-2 (**Supplementary Figure S9**). The observed overlap between maltreatment reported at wave-1 and wave-2 may intersect, and wave-1 data may confound the findings at wave-2 on alcohol consumption (**Supplementary Figure S9**). Outcome-intersection means the intersection of several mental health outcomes, possibly stemming from a single intermediate phenotype, such as a deficit in emotion regulation (94). For example, we observed that outcomes such as high alcohol drinking co-occur with other outcomes such as nicotine use, illicit drug use, and delinquency (**Tables 1**, **2**). In both predictor and outcome intersections, isolating the effect of a single predictor or outcome is challenging, respectively. We handled the predictor-intersection problem by adjusting the significant three-way interactions for co-occurring and re-occurring environmental factors to isolate the effect of single maltreatment. The outcome intersection was illustrated by adjusting the three-way interactions to co-occurring and re-occurring behavioural outcomes to isolate the effect of alcohol consumption. Using the “all-in-one” approach, both the three-way interactions with the FM and NFM were non-significant, but this could probably be due to the overfitting of the model (**Supplementary Tables S8A**, **S10A**). However, using the single approach, we observed that the three-way interaction with NFM was robust to all the intersecting predictor environmental and behavioural covariates, except for the parent’s alcohol use, negative parent-child relationship and adolescents’ nicotine use reported at wave-2 (**Supplementary Tables S8B**, **S10B**). The findings indicate that having a good parent-child relationship protects robustly against the negative effect of NFM on alcohol consumption in *MAOA*-L allele-carrying females. Another way to handle the predictor intersection problem could be to create a composite variable to merge all the potential predictors into one. However, we did not choose it due to our goal of characterizing the sample. Moreover, the outcome intersection may obscure potential G×E associations, and thus, co-occurring outcomes like substance use and antisocial outcomes should be considered together with alcohol consumption in future G×E studies.

Indeed, alcohol use disorder is a genetically complex psychiatric disorder which includes genetic and neurobiological heterogeneity, polygenicity, and interaction of genes with the environment (95). Thousands of genes and their common and rare variants with small effect sizes contribute to the complexity of AUD. However, identifying the contribution of a single candidate gene (*MAOA*), selected on the basis of biological plausibility, despite its small effect size, is important to understand the small part of the genetic puzzle of AUD. Our group has studied MAOA in interaction with environment in four cohorts and have found relatively good explained variation in the model of alcohol consumption (*MAOA*-uVNTR × poor family relation or maltreatment/abuse/sexual abuse explained 23-25% variation in alcohol consumption and alcohol-related problematic behaviour among 16- and -19 year Swedish adolescent females (60), *MAOA*-uVNTR × poor family relations or sexual abuse explained 7-10% variance in alcohol consumption among Swedish 17-18 year adolescent males and females (58), Interaction of *MAOA*-uVNTR with maltreatment moderated by *MAOA* DNA methylation explained 9.4% variance in alcohol consumption among mean age 22.1 year old Swedish young adult males, who sought treatment for substance misuse (72), and in the present study, interaction of *MAOA*-uVNTR × Family or non-family maltreatment moderated by positive parent-child relationship explained 5 to 6% variation in alcohol consumption among Swedish 16-19 year adolescent females). Replication of the association of *MAOA*-uVNTR with alcohol consumption in interaction with negative and/or positive environment by our present and previous findings together with others (61, 62, 79), strengthens the individual contribution of *MAOA*-uVNTR, not alone but in interaction with the environment, to explain a complex polygenic trait of alcohol use disorder. Although a single locus might not fully explain the heterogenous and polygenic trait, the interplay between the specific genetic variants and environmental factors can provide additional predictive power. Indeed, given the role of the MAOA gene in encoding the MAOA enzyme that metabolizes monoaminergic neurotransmitters involved in mediating reward, polymorphisms in the genes in dopaminergic (DRD2, DRD4 and DAT1 genotypes), or serotonergic (5-HTTLPR) neurotransmission cumulatively in the interaction of environment could explain more variation in the alcohol consumption and can better predict the risk of alcohol consumption (66, 96). However, these candidate genes have been first individually associated with alcohol consumption and related phenotypes in interaction with either a negative or positive environment [see review (97)].

Alternative to using genetic risk or plasticity scores on the basis of a single or combination of several candidate genes, Genome-Wide Association Studies (GWAS) derived polygenic scores emerged in gene-environment research to improve the prediction accuracy of complex traits by considering the cumulative impact of multiple loci. Polygenic scores are based on SNPs typically linked to multiple genes across the genome that may collectively influence the trait. Despite hundreds of genes, all measured SNPs together typically explain not more than 10% variances in substance use (98); specifically, polygenic scores explained around 1.9% variance in alcohol consumption (99) or <0.1% variance in alcohol consumption upon interaction with the environment (100). However, as recently pointed out by Zhang and Belsky (97), it is important to note that i) GWAS derived polygenic scores are exclusively based on SNPs and do not include any other types of genetic polymorphisms such as variable number tandem repeats as in *MAOA* gene, thereby providing limited information to G×E research, ii) These are hypothesis free and polygenic score are derived on the basis of mere correlation of SNPs with phenotype and not on the basis of biological plausibility, thereby making predictions of complex traits and replication of results difficult in samples with heterogenous phenotypes iii) SNPs identified in GWAS are not the true causal elements but are proxies that correlate with the true causal variants, whereas, the in case of variable number tandem repeats, the risk allele itself is considered as the causal element, iv) GWAS-derived polygenic score are sensitive to variation in racial and ethnic patterns of linkage disequilibrium and therefore, polygenic scores based on one race or ethnicity may be difficult to use in another. For example, the results from a recent G×E study that predicted alcohol use among adults using polygenic scores composed merely of European ancestry may not be generalizable to other ancestry (99). In addition, the G×E studies with polygenic scores are still weak to robustly predict complex traits like substance use (98), and they are not suitable for G×E inquiries testing differential susceptibility hypothesis because the SNP having a main effect on the phenotype are included in polygenic risk scores and not the SNPs that have an effect on differential susceptibility, as they may not have large effects to be detected in GWAS (101).

The strengths of the present study are: 1) Large sample size- which gave us adequate power to detect moderate to large effect size (*MAOA×* FM×positive parent-child relationship: *R^2^* increase = 0.5%, observed power = 0.541; and *MAOA*×NFM×positive parent-child relationship: *R^2^* increase = 1%, observed power = 0.832). Large sample size also helped us overcome the problem of false-negative/positive findings observed in small sample-sized G×E studies (102). 2) We used a differential susceptibility framework incorporating a more holistic range of risk and protective environmental factors than older studies, which often overlook factors mitigating the risk. 3) Collecting maltreatment self-reports at two-time points allowed us to account for instances of maltreatment that participants were hesitant to report earlier. 4) The sample was representative of a segment of the general adolescent population, leading to the generalizability of the findings to a non-clinical, non-refereed population. The limitations of the study are 1) Reliability on retrospective self-reports, which involve a risk of recall bias. 2) As we measured the parent-child relationship only at a one-time point, we could not establish a causal relationship. 3) Using an at-home-based postal questionnaire, presumably overseen by the parent, may have prevented the adolescents from the most adverse social environments from participating in the present study. 4) The association of cG×Eneg×Epos with alcohol consumption in females carrying the non-risk allele might not be accurately represented due to a small sample size (**Figures 2**, **3**). Therefore, the findings in females carrying the non-risk allele should be cautiously interpreted and need replication in larger samples.

## Conclusions

The present study provides the first evidence that the MAOA-L allele (SL/LL genotype) in females is sensitive to negative and positive environments concerning their alcohol use. Furthermore, the study indicates that a good parent-child relationship, especially the warmth dimension, protects against alcohol consumption among the MAOA-L allele-carrying females exposed to maltreatment. However, the interactions were not significant after adjusting to several environmental and behavioural covariates, especially parent’s alcohol use, negative parent-child relationship, and nicotine use (smoking and/or snus) suggesting predictor and outcome intersection. Thus, the results indicate that alcohol use and nicotine use are intercorrelated and that future studies and frameworks for preventive strategies should target these co-occurring behaviours together. Insights into differential susceptibility of the MAOA-L allele to the environment may help to explain the lack of association between *in-vivo* and *in-vitro* allele-specific transcriptional activity. The study underscores the need to re-evaluate the diathesis-stress-based MAOA×environment research strategy and recommends including negative and positive environments in the analyses. The study also emphasizes the importance of considering early life events and parent-child relationships to design personalized intervention strategies for AUD. The study calls for future studies to effectively assess and address the predictor- and outcome-intersection phenomenon. As it is the first study to address differential susceptibility of MAOA-uVNTR concerning alcohol use, replication in larger samples and molecular research assessing underlying epigenetic mechanisms will be beneficial for future MAOA×E studies regarding alcohol use.

## Data availability statement

The raw data supporting the conclusions of this article will be made available by the authors upon request, without undue reservation.

## Ethics statement

The studies involving humans were approved by Regional Board for Research Ethics in Uppsala, Sweden (Ref: 2012/187). The studies were conducted in accordance with the local legislation and institutional requirements. Written informed consent for participation in this study was provided by the participants’ legal guardians/next of kin.

## Author contributions

MB: Conceptualization, Formal Analysis, Investigation, Methodology, Validation, Visualization, Writing – original draft, Writing – review & editing. DC: Methodology, Writing – review & editing. AT: Writing – review & editing. CA: Conceptualization, Formal Analysis, Funding acquisition, Investigation, Methodology, Validation, Writing – review & editing. SH: Methodology, Writing – review & editing, Formal Analysis. KN: Conceptualization, Funding acquisition, Investigation, Methodology, Project administration, Resources, Supervision, Writing – review & editing.
