# Supplementary material for: Good parent-child relationship protects against alcohol use in maltreated adolescent females carrying the MAOA-uVNTR susceptibility allele
Source: Front Psychiatry. 2024 Jul 22;15:1375363. doi: 10.3389/fpsyt.2024.1375363 (PMC11298380; doi:10.3389/fpsyt.2024.1375363)
Supplement: Supplementary file 1 [file DataSheet_1.docx]

Supplementary Material

**Good parent-child relationship protects against alcohol use in maltreated adolescent females carrying the *MAOA*-uVNTR susceptibility allele**

#### Material and Methods

##### Positive Parent-child relationship

The positive dimensions outlined in PASCQ (1) included three dimensions; **warmth, structure, and** **autonomy support**. Dimension **warmth** included four statements: 1) My parents show that they love me, 2) My parents like to be with me, 3) My parents are always happy to see me, and 4) I am special and important to my parents. Dimension **Structure** included four statements: 1) When I want to do something, my parents show me how, 2) When I want to understand how something works, my parents explain it to me, 3) If I have a problem, my parents will help me figure out how to solve it, and 4) My parents explain why we have our family rules. Dimension **Autonomy support** included four statements: 1) My parents trust me, 2) My parents accept me as I am, 3) My parents let me do the things that I think are important, and 4) My parents try to understand my point of view. For responses to these statements see materials and methods in the main text.

##### Covariates

We adjusted the significant three-way interactions to the following covariates accounted at either wave-1/wave-2 or at both the waves.

##### Negative Parent-child relationship

The negative aspect of the parent-child relationship (1) was reported at wave-2 only and included three dimensions; **Rejection** (Sometimes I wonder if my parents like me, My parents think I'm always in the way, My parents make me feel like I'm not wanted, and nothing I do is good enough for my parents.), **Chaos** (When my parents promise something, I never know if they will keep it; When my parents say they are going to do something, it does not happen at all; My parents change the rules for me all the time; and, My parents get angry with me without warning.), **Coercion** (My parents always tell me what to do, My parents even settle over me, My parents think there's one way to do things - their way, and, My parents say "no" to everything). Participants rated each statement on a scale of 0˗3 (0 = Not at all true, 1= Not very true, 2= sort of true, and 3= Very true (Cronbach's alpha = 0.744). A scale for a negative relationship with parents was computed as the sum of 12 statements comprising Rejection, Chaos, and Coercion, which ranged from 0 to 36, with higher scores indicating an increasingly negative relationship with parents. In order to compute a composite covariate variable based on the principal component analysis (details in material and methods under posthoc analyses in the main text) which was then used in “all-in-one” approach in posthoc analyses, we created a binary variable, where, negative relationship scores= mean or one standard deviation below mean (i.e score from 0-7) were coded as 0, and score ≥ Mean +1SD (i.e score from 8-36) were coded as 1.

##### Nicotine use

Nicotine use was defined as the use of any nicotine-containing products, such as cigarettes or moist snus, and was reported at wave-1 or -2. The variable was binary coded into users (coded as 1) and non-users (coded as 0). Participants were grouped into "users" if they reported the use of nicotine-containing products sometimes or daily, whereas they were grouped into "non-users" if they never or have quit using nicotine-containing products. We also used this binary coded variable to compute a composite covariate variable based on the principal component analysis (details in material and methods under posthoc analyses in the main text) to adjust the three-way interactions using in “all-in-one” approach in posthoc analyses.

##### Use of illicit drugs

Data on the use of illicit drugs was collected only at Wave-1 (Mean age:14.4 yeas). Participants were asked if they used: 1) hash/marijuana and/ or 2) any drugs other than hash/marijuana in life. Participants reported on the frequency of using each of these drugs and were coded as 0 = Never, 1 = Yes, one time, 2 = Yes, 2-4 times, 3 = Yes, 5-10 times, 4 = Yes, 11-20 times, 5 = Yes, 21-50 times, and (6) Yes, more than 50 times. The sum of the two questions was used as an index of the use of illicit drugs (ranged 0 to 12). The highest score indicated a high frequency of using illicit drugs. For the descriptive purpose, the variable was binary coded, where those who never used illicit drugs were coded as 0, and those who used it one or more times were coded as 1. We also used this binary coded variable to compute a composite covariate variable based on the principal component analysis (details in material and methods under posthoc analyses in the main text) to adjust the three-way interactions using in “all-in-one” approach in posthoc analyses.

##### Bullying in school

To account for bullying in school, we used an in-house questionnaire. At both wave-1 and wave-2, participants were asked if they experienced the following five events in school during the study year. If they experienced the said event, then the participants reported its frequency. The questions included: Q1) Have you during this past year been beaten, kicked or physically abused by anybody in your school? Q2) Have you severely been provoked, for example, being accused of things you have not done, been threatened, or been called names by anybody in your school this past year? Q3) Has anybody during this past year in school lied about you, talked about you behind your back, ignored you or alienated you? Q4) Have you been subjected to bullying, threats or harassment via mobile phone and/or internet? Answers were coded as 0 = Never, 1 = yes, some or many times in a year, 2 = yes, 1 to 3 times in a month, 3 = Yes, at least once in a week. The sum of the four questions was used as an index of bullying in school (ranged 0 to 12). The highest score indicated a high frequency of bullying in school. For the descriptive purpose, the variable was binary coded, where those who never experienced bullying in school were coded as 0, and those who experienced bullying in school were coded as 1. We also used this binary coded variable to compute a composite covariate variable based on the principal component analysis (details in material and methods under posthoc analyses in the main text) to adjust the three-way interactions using in “all-in-one” approach in posthoc analyses.

##### Delinquency

Participants at wave-1 and 2 were reported on their delinquent behaviour using an in-house questionnaire adapted from a measure of conduct-problems outlined by Andershed and co-workers (2). The questionnaire included twenty-three questions regarding property offences, violent offences towards animals and other people, vandalism, and coercive behaviours. Responses were coded using a five-point scale that included and were coded as; (0) "No, that has not happened", (1) "1 time", (2) "2-3 times", (3) "4-10 times", and (4) "More than ten times". Of the 23 items, 15 items referred to non-violet- and eight items referred to violent- delinquent behaviour. Two composite variables were computed; first, the non-violent- delinquent behaviour measure included the sum of 15 items with a score range 0-60, and second, the violent- delinquent behaviour measure included the sum of 8 items with a score range 0-32. A higher score indicated a higher frequency of delinquent behaviour. In order to compute a composite covariate variable based on the principal component analysis (details in material and methods under posthoc analyses in the main text) which was then used in “all-in-one” approach in posthoc analyses, we created a binary variable for non-violent- and delinquent behaviours, where, score=0 was coded as 0, and any score above 0 was coded as 1.

##### Parent's alcohol use

At wave-1, parents were asked to report on their alcohol use by responding to the same three items as the adolescents with a score ranging from 0 to 14. A higher score indicated higher alcohol drinking behaviour. In total 98.6% (n = 1397) adolescent’s parents reported their alcohol use. In order to compute a composite covariate variable based on the principal component analysis (details in material and methods under posthoc analyses in the main text) which was then used in “all-in-one” approach in posthoc analyses, we created a binary variable, where, parents’ AUDIT-C score=0 was coded as 0, and any score above 0 was coded as 1.

##### Positive and negative life events

At wave-2, life events in the last 12 months were accounted for using a checklist for 40 events, child and adolescent survey of experiences (CASE) (3). Participants rated the impact of the reported event on a 6-point scale from 1 (really good), 2 (quite good), 3 (little good), 4 (little bad), 5 (quite bad), to 6 (really bad). An event was considered as positive if the impact of the event was rated as either "really good" or "quite good" or "little good", while an event was considered as negative if the impact of the event was rated as either "really bad" or "quite bad" or "little bad". The total number of positive and negative life events were counted to create two separate variables called "Total number of positive life events (PLE)" and "Total number of negative life events (NLE)", each variable ranged from 0 to 40. In order to compute a composite covariate variable based on the principal component analysis (details in material and methods under posthoc analyses in the main text) which was then used in “all-in-one” approach in posthoc analyses, we created a binary variable on the basis of median, where, PLE or NLE scores=0-5 were coded as 0, and PLE or NLE scores from 6-40 were coded as 1.

#### S1 Table. List of the variables used as covariates in the model for AUDIT-C at wave 2 containing three-way interaction terms.

| **Variables measured at wave 1** | **Variables measured at wave 2** |
| --- | --- |
| Adolescents’ AUDIT-C score^1^ |  |
| Parents’ AUDIT-C score^1^ |  |
| Use of illicit substances^1^ |  |
|  | Negative parent-child relationship^1^ |
|  | Positive life events^1^ |
|  | Negative life events^1^ |
| Nicotine use^1^ | Nicotine use^1^ |
| Bullying in school^1^ | Bullying in school^1^ |
| Family maltreatment (FM)^2,3^ | Family maltreatment (FM)^3^ |
| Non-family maltreatment (NFM)^2,3^ | Non-family maltreatment (NFM)^2^ |
| Violent delinquency^1^ | Violent delinquency ^1^ |
| Non-Violent delinquency ^1^ | Non-Violent delinquency ^1^ |

^1^ Covariate in both the models of AUDIT-C (wave -2) containing: a) *MAOA*×FM (wave-2)×positive parent-child relationship term, and b) *MAOA*×NFM (wave-2)× positive parent-child relationship term.

^2^Covariate in the model of AUDIT-C (wave -2) containing MAOA×FM (wave-2)×positive parent-child relationship term.

^3^Covariate in the model of AUDIT-C (wave -2) containing MAOA×NFM (wave-2)×positive parent-child relationship term.

#### S2 Table. Principal component analysis on covariates.

| **Covariate** | **Component 1** | **Component 2** | **Component 3^a^** | **Component 4 ^a^** | **Component 5** |
| --- | --- | --- | --- | --- | --- |
| Violent Delinquency, Wave-1 | 0.79 |  |  |  |  |
| Violent Delinquency, Wave-2 | 0.684 |  |  |  |  |
| Non-violent Delinquency, Wave-1 | 0.679 |  |  |  |  |
| Use of illicit substances, Wave-1 | 0.658 |  |  |  |  |
| Non-violent Delinquency, Wave-2 | 0.575 |  |  |  |  |
| AUDIT-C, Wave-1 |  | 0.779 |  |  |  |
| Nicotine use, Wave-1 |  | 0.776 |  |  |  |
| Nicotine use, Wave-2 |  | 0.642 |  |  |  |
| Non-family maltreatment, Wave-2^b^ |  |  | 0.695 |  |  |
| Bullying in school, Wave-2 |  |  | 0.674 |  |  |
| Bullying in school, Wave-1 |  |  | 0.629 |  |  |
| Non-family maltreatment, Wave-1 |  |  | 0.624 |  |  |
| Family maltreatment, Wave-2^c^ |  |  |  | 0.843 |  |
| Negative parent-child relationship |  |  |  | 0.753 |  |
| Family maltreatment, Wave-1 |  |  |  | 0.662 |  |
| Negative life events |  |  | 0.402 | 0.439 |  |
| Positive life events |  |  |  |  | 0.709 |
| AUDIT-C parent |  |  |  |  | 0.442 |
| Eigen values | 4.938 | 2.094 | 1.220 | 1.144 |  |
| % of variance explained | 27.435 | 11.632 | 6.780 | 6.356 | 6.254 |
| Composite variable name | Behavioural problems 1 | Behavioural  problems 2 | Other negative environmental factors | | Parent's alcohol use and Positive life events |

| Extraction Method: Principal Component Analysis. |
| --- |
| Rotation Method: Varimax with Kaiser Normalization. |

^a^Covariates in component 3 and 4 combined together to form one composite variable called “Other negative environmental factors” because it included all the negative environmental factors and explained almost similar variance.

^b^Included in the composite variable called “Other negative environmental factors” for the model of AUDIT-Cw2 with *MAOA*-uVNTR genotype × FM × Positive parent-child relationship term.

^C^Included in the composite variable called “Other negative environmental factors” for the model of AUDIT-Cw2 with *MAOA*-uVNTR genotype × NFM × Positive parent-child relationship term.

#### S3 Table. Family characteristics of the sample.

| **Family characteristics** | **Males**  **(*N*,%)** | **Females (*N*,%)** | ***χ*^2^**  **(*p*-value)** |
| --- | --- | --- | --- |
| **With whom do you live?** |  |  |  |
| Biological Mother, Biological father, and siblings | 302 (53.2) | 444 (52.4) | 25.398 (0.552) |
| Biological Mother and siblings | 19 (3.3) | 34 (4.0) |  |
| Biological father and siblings | 1 (0.2) | 3 (0.4) |  |
| Biological Mother only | 15 (2.6) | 38 (4.5) |  |
| Biological Father only | 2 (0.4) | 5 (0.6) |  |
| Biological Mother, Biological father | 105 (18.5) | 138 (16.3) |  |
| Biological mother, bonus/step father, and siblings | 20 (3.5) | 27 (3.2) |  |
| Biological father, bonus/step mother, and siblings | 14 (2.5) | 5 (0.6) |  |
| Biological mother and bonus/step father | 2 (0.4) | 20 (2.4) |  |
| Biological father and bonus/step mother | 41 (7.2) | 3 (0.4) |  |
| Biological Mother, Biological father, siblings and Bonus/Step mother | 1 (0.2) | 4 (0.5) |  |
| Biological Mother, Biological father, siblings and Bonus/Step Father | 4 (0.7) | 3 (0.4) |  |
| Biological Mother, Biological father, siblings, Bonus/Step Father and mother | 2(0.4) | 3 (0.4) |  |
| Biological Mother, Biological father, siblings but sometime with mumma/pappa | 0 | 2 (0.2) |  |
| Biological Mother, Biological father, siblings, sometime with mumma/pappa, and bonus/step mother | 0 | 2 (0.2) |  |
| Biological Mother, Biological father, siblings, sometime with mumma/pappa, bonus/step mother and father | 0 | 3 (0.4) |  |
| Sometimes with Biological Mother or Biological Father, and siblings | 12 (2.1) | 18 (2.1) |  |
| Sometimes with Biological Mother or Biological Father, siblings, and bonus/step mother | 3 (0.5) | 8 (0.9) |  |
| Sometimes with Biological Mother or Biological Father, siblings, and bonus/step father | 2 (0.4) | 6 (0.7) |  |
| Sometimes with Biological Mother or Biological Father and siblings and bonus/step mother and father | 5 (0.9) | 10 (1.2) |  |
| Sometimes with Biological Mother or Biological Father | 3 (0.5) | 55 (6.5) |  |
| Sometimes with Biological Mother or Biological Father, and bonus/step father | 2 (0.4) | 2 (0.2) |  |
| Sometimes with Biological Mother or Biological Father, and bonus/step mother | 3 (0.5) | 3 (0.4) |  |
| Sometimes with Biological Mother or Biological Father, and bonus/step mother and bonus/step father | 1 (0.2) | 3 (0.4) |  |
| Alone or with boyfriend or girlfriend | 2(0.4) | 1 (0.1) |  |
| Foster family | 6 (1.1) | 6 (0.7) |  |
| Missing family data | 1 (0.2) | 2 (0.2) |  |
| **Parents marital status** | | | |
| Separated parents | 528 (31.4) | 221 (30.6) | 1.821 (0.177) |
| Non-separated parents | 1127 (67.0) | 487 (67.5) |  |
| **Parents working status** | | | |
| Mother working or studying | 1675 (99.6) | 717 (99.3) | NA |
| Father working or studying | 1675 (99.6) | 717 (99.3) | NA |

NA: Not applicable because the variable is constant.

#### S4 Table. Characteristics of male and female high and low alcohol drinkers^1^ on main measures in the study reported at wave-2.

| **a) Main measures (Categorical variables)** | **HDM**  ***n* (%)** | **LDM**  ***n* (%)** | **HDM vs**  **LDM**  *χ*^2^ | **HDF**  ***n* (%)** | | **LDF**  ***n* (%)** | | **HDF vs**  **LDF**  *χ*^2^ | | **HDM vs**  **HDF**  *χ*^2^ | | **LDM vs**  **LDF**  *χ*^2^ | |  |
| --- | --- | --- | --- | --- | --- | --- | --- | --- | --- | --- | --- | --- | --- | --- |
| Sample size | 88 (15.5) | 480 (84.5) |  | 214 (25.2) | | 634 (74.5) | |  | |  | |  | |  |
| *Genetic factor* |  |  |  |  | |  | |  | |  | |  | |  |
| *MAOA-S* | 37 (42.0) | 183 (38.1) | 0.48 |  | |  | |  | |  | |  | |  |
| *MAOA-L* | 51 (58.0) | 297 (61.9) |  |  | |  | |  | |  | |  | |  |
| *MAOA-SS* |  |  |  | 22 (10.3) | | 81 (12.8) | | 0.93 | |  | |  | |  |
| *MAOA-SL/LL* |  |  |  | 192 (89.7) | | 553 (87.2) | |  | |  | |  | |  |
| *Negative environmental factors* |  |  |  |  | |  | |  | |  | |  | |  |
| *FM wave-2* | 41 (46.6) | 207 (43.1) | 0.36 | 123 (57.5) | | 304 (47.9) | | 5.81* | | 2.978 | | 2.44 | |  |
| Witnessed psychological maltreatment between parents | 37 (42.0) | 191 (39.8) | 0.16 | 110 (51.4) | | 273 (43.1) | | 4.50* | | 2.19 | | 1.11 | |  |
| Witnessed physical maltreatment between parents | 4 (4.5) | 14 (2.9) | 0.64 | 19 (8.9) | | 23 (3.6) | | 9.34* | | 1.66 | | 0.45 | |  |
| Experienced psychological maltreatment by parents | 11 (12.5) | 48 (10.0) | 0.50 | 53 (24.8) | | 104 (16.4) | | 7.42* | | 5.62* | | 9.59* | |  |
| Experienced physical maltreatment by parents | 8 (9.1) | 34 (7.1) | 0.44 | 31 (14.5) | | 42 (6.6) | | 12.57* | | 1.61 | | 0.09 | |  |
| *NFM wave-2* | 29 (33.0) | 88 (18.3) | 9.65* | 82 (38.3) | | 139(21.9) | | 22.31* | | 0.77 | | 1.92 | |  |
| Experienced psychological maltreatment by a non-family adult | 24 (27.3) | 83 (17.3) | 4.80* | 81 (37.9) | | 139 (21.9) | | 21.12* | | 3.08 | | 3.34 | |  |
| Experienced physical maltreatment by a non-family adult | 15 (17.0) | 21 (4.4) | 19.98* | 20 (9.3) | | 17 (2.7) | | 16.93* | | 3.61 | | 2.37 | |  |
| *Positive environmental factors* |  |  |  |  | |  | |  | |  | |  | |  |
| *Positive parent-child relationship, wave-2* |  |  |  |  | |  | |  | |  | |  | |  |
| Poor | 18(20.5) | 86(17.9) | 0.33 | 46 (21.5) | | 84 (13.2) | | 10.00* | | 0.22 | | 5.04 | |  |
| Average | 23 (26.1) | 127 (26.5) |  | 60 (28.0) | | 167 (26.3) | |  |  |  |  |  |  |  |
| Good | 47 (53.4) | 267 (55.6) |  | 108 (50.5) | | 383 (60.4) | |  |  |  |  |  |  |  |
| **b) Main measures (Ordinal variables)** | **HDM**  **Median**  **(Min-Max)** | **LDM**  **Median**  **(Min-Max)** | **HDM vs LDM**  ***U*** | **HDF**  **Median**  **(Min-Max)** | | **LDF**  **Median**  **(Min-Max)** | | **HDF vs LDF**  ***U*** | | **HDM vs HDF**  ***U*** | | **LDM vs LDF**  ***U*** | |  |
| *Age* | 18.2  (15.8-18.8) | 16.7  (15.8-18.8) | 10853.5* | 17.9 (0.9) | | 17.2 (1.0) | | 43546.5* | | 8068.5* | | 151149.0 | |  |
| *FM score, wave-2* | 0 (0-12) | 0 (0-11) | 20857.0 | 1 (0-12) | | 0 (0-12) | | 57619.0* | | 7508.0* | | 142529.5* | |  |
| *NFM score, wave-2* | 0 (0-6) | 0 (0-5) | 17796.5* | 0 (0-6) | | 0 (0-6) | | 55667.5* | | 8982.0 | | 146843.0 | |  |
| *Positive parent-child relationship score, wave-2* | 29 (3-36) | 29 (9-36) | 19825.5 | | 29 (9-36) | | 30 (5-36) | | 57831.0* | | 9349.5 | | 138868.5* | |

^1^ For cut-off for high-risk alcohol consumption see materials and methods.

^2^ Categories based on Mean ± 1SD.

**p*-values (2-sided asymptotic significance) ≤ 0.05

FM: Family maltreatment; HDF: High-alcohol drinking females; HDM: High-alcohol drinking males; LDF: Low-alcohol drinking females; LDM: Low-alcohol drinking males; *MAOA*: Monoamine oxidase A; NFM: Non-family maltreatment.

#### S5 Table. Characteristics of male and female high and low alcohol drinkers^1^ on covariates in the study.

| 1. **Covariates (Categorical variables)** | **HDM**  ***N* (%)** | **LDM**  ***N* (%)** | **HDM vs LDM,** *χ*^2^ | **HDF**  ***N* (%)** | **LDF**  ***N* (%)** | **HDF vs LDF,** *χ*^2^ | **HDM vs HDF,** *χ*^2^ | **LDM vs LDF,** *χ*^2^ |
| --- | --- | --- | --- | --- | --- | --- | --- | --- |
| Sample size | 88 (15.5) | 480 (84.5) |  | 214 (25.2) | 634 (74.5) |  |  |  |
| **Environmental factors** | |  |  |  |  |  |  |  |
| *FM, Wave-1* | 27(30.7) | 130 (27.1) | 0.62 | 81 (37.9) | 160 (25.2) | 12.09* | 1.088 | 0.48 |
| Witnessed psychological maltreatment between parents | 20 (23.8) | 109 (23.2) | 0.02 | 73 (34.1) | 137 (21.6) | 12.92* | 3.15 | 0.50 |
| Witnessed physical maltreatment between parents | 1 (1.2) | 8 (1.7) | 0.12 | 14 (6.5) | 11 (1.7) | 12.71* | 3.73* | 0.01 |
| Experienced psychological maltreatment by parents | 7 (8.2) | 29 (6.2) | 0.51 | 34 (15.9) | 42 (6.6) | 16.52* | 3.10 | 0.16 |
| Experienced physical maltreatment by parents | 6 (7.1) | 28 (5.9) | 0.16 | 26 (12.1) | 26 (4.1) | 17.73* | 1.71 | 0.18 |
| *NFM, wave-1* | 12(13.6) | 44(9.4) | 1.92 | 30 (14.1) | 61 (9.7) | 3.07 | 0.00 | 0.07 |
| Experienced psychological maltreatment by a non-family adult | 11 (12.5) | 40 (8.3) | 1.81 | 27 (12.7) | 58 (9.1) | 2.01 | 0.01 | 0.24 |
| Experienced physical maltreatment by a non-family adult | 4 (4.6) | 12 (2.5) | 1.20 | 4 (1.9) | 11(1.8) | 0.01 | 1.84 | 0.79 |
| *Negative parent-child relationship, wave-2* |  |  |  |  |  |  |  |  |
| Low | 15 (17.0)  36 (40.9)  37 (42.1) | 71 (14.8) | 0.41 | 34 (15.9) | 107 (16.9) | 1.88 | 0.99 | 2.03 |
| Average |  | 210 (43.8) |  | 83 (38.8) | 255 (40.2) |  |  |  |
| High |  | 199 (41.5) |  | 97 (45.3) | 272 (42.9) |  |  |  |
| *Bullied in school, wave-1* | 31 (35.2) | 151 (31.5) | 0.49 | 117 (54.7) | 248 (39.1) | 15.79* | 9.44* | 6.89* |
| *Bullied in school, wave-2* | 27 (30.7) | 126 (26.3) | 0.74 | 115 (53.7) | 282 (44.5) | 5.51* | 13.31* | 38.85* |
| *Parents with alcohol use* | 82 (93.18) | 398 (82.92) | 5.99* | 194 (90.7) | 532 (83.9) | 5.91* | 0.51 | 0.21 |
| ***Behavioral factors*** |  |  |  |  |  |  |  |  |
| *Nicotine users, wave-1* | 14 (15.9) | 15 (3.1) | 25.27* | 38 (17.8) | 12 (1.9) | 73.45* | 0.13 | 1.85 |
| *Nicotine users, wave-2* | 58 (65.9) | 76 (15.8) | 103.45* | 115 (53.7) | 59 (9.3) | 193.68 | 3.78* | 10.91* |
| *Illicit drug users, wave-1* | 1 (1.1) | 3 (0.6) | 0.28 | 2 (0.9) | 1 (0.2) | 2.76 | 0.03 | 1.71 |
| *Involved in non-violent delinquent behaviors, wave-1* | 58 (65.90) | 226 (47.08) | 10.54* | 144 (67.3) | 220 (34.7) | 69.36* | 0.05 | 17.95* |
| *Involved in non-violent delinquent behaviors, wave-2* | 73 (83.0) | 262 (54.6) | 24.74* | 156 (72.9) | 303 (47.8) | 40.61* | 3.44 | 5.31* |
| *Involved in violent delinquent behaviors, wave-1* | 43 (48.9) | 165 (34.4) | 6.73* | 44 (20.6) | 56 (8.8) | 21.16* | 24.36* | 113.37* |
| *Involved in violent delinquent behaviors, wave-2* | 51 (58.0) | 169 (35.2) | 16.21* | 56 (26.2) | 79 (12.5) | 22.46* | 27.54* | 81.45* |
| 1. **Covariates (Ordinal variables)** | **HDM**  **Median**  **(Min–Max)** | **LDM**  **Median**  **(Min–Max)** | **HDM**  **vs LDM,** *U* | **HDF**  **Median**  **(Min–Max)** | **LDF**  **Median**  **(Min–Max)** | **HDF vs LDF,**  *U* | **HDM vs HDF,**  *U* | **LDM vs LDF,**  *U* |
|  |  |  |  |  |  |  |  |  |
| AUDIT-C scores, wave-1 | 0 (0-10) | 0 (0-11) | 14861.5* | 0 (0-11) | 0 (0-7) | 44468.0* | 8462.0 | 145949.0 |
| FM score, Wave-1 | 0 (0-5) | 0 (0-9) | 19510.0 | 0 (0-12) | 0 (0-13) | 56195.0* | 8003.0 | 143989.0 |
| NFM score, wave-1 | 0 (0-4) | 0 (0-5) | 19046.5 | 0 (0-5) | 0 (0-5) | 63138.0 | 8995.0 | 146147.0 |
| Negative parent-child relationship score, wave-2 | 7 (0-30) | 7 (0-28) | 20710.5 | 7 (0-33) | 7 (0-28) | 66030.5 | 9211.0 | 149962.0 |
| Number of positive life events, wave-2 | 6 (0-15) | 6 (0-16) | 17896.0* | 6 (0-15) | 6 (0-21) | 62235.0 | 9319.0 | 138627.0 |
| Number of negative life events, wave-2 | 3 (0-17) | 1 (0-13) | 15239.5* | 4 (0-20) | 2 (0-18) | 48565.5* | 7711.5* | 116559.5* |
| Frequency of bullying in school, wave-1 | 0 (0-9) | 0 (0-8) | 19532.5 | 1 (0-9) | 0 (0-9) | 54545.0* | 6904.5* | 135009.5* |
| Frequency of bullying in school, wave-2 | 0 (0-4) | 0 (0-5) | 19723.5 | 1 (0-12) | 0 (0-12) | 59662.5* | 7130.5* | 123350.0 |
| Parent’s AUDIT-C score | 4 (0-10) | 4 (0-12) | 16293.0* | 4 (0-9) | 4 (0-12) | 56659.5* | 8372.5 | 144322.0 |
| Frequency of Illicit drug use, wave-1 | 2 (2-11) | 2 (1-7) | 19529.5* | 2 (2-11) | 2 (1-6) | 64057.0* | 9079.5 | 147028.0 |
| Non-violent delinquency score, wave-1 | 2 (0-30) | 0 (0-26) | 15464.5* | 2 (0-30) | 0 (0-12) | 39096.5* | 9060.5 | 127779.0 |
| Non-violent delinquency score, wave-2 | 4 (0-44) | 1 (0-29) | 12327.0* | 2 (0-31) | 0 (0-30) | 41730.5* | 7542.0* | 132176.0* |
| Violent delinquency score, wave-1 | 0 (0-14) | 0 (0-12) | 17357.5* | 0 (0-14) | 0 (0-5) | 59744.0* | 6627.0* | 112824.0* |
| Violent delinquency score, wave-2 | 1 (0-22) | 0 (0-12) | 15361.0* | 0 (0-12) | 0 (0-16) | 58064.5* | 6213.0* | 116541.0* |

^1^ Cut-off for high-risk alcohol consumption is according to Nilsson et al.,2011 (4) and is based on wave-2 AUDIT-C scores.

^2^ Categories are based on Mean ± 1SD.

^*^ Asymptomatic *p*-value (2-sided) ≤ 0.05.

FM: Family maltreatment; HDF: High alcohol drinking females; HDM: High alcohol drinking males; LDF: Low alcohol drinking females; LDM: Low alcohol drinking males; MAOA: Monoamine oxidase A; NFM: Non-family maltreatment.

#### S6 Table. Bivariate correlations between main variables reported at wave-1 and/or wave-2, in the total sample as well as in males and females.

| **Bivariate correlations** | **Total sample** | **Males** | **Females** |
| --- | --- | --- | --- |
|  | ***r_s_*** | ***r_s_*** | ***r_s_*** |
| **Wave-1** | | | |
| AUDIT-C wave-1 and FM wave-1 | 0.194** | 0.178** | 0.203** |
| AUDIT-C wave-1 and NFM wave-1 | 0.145** | 0.135* | 0.150** |
| FM wave-1 and NFM wave-1 | 193** | 0.161** | 0.213** |
| **Wave-2** | | | |
| AUDIT-C wave-2 and FM wave-2 | 0.082* | 0.009^a^ | 0.134** |
| AUDIT-C wave-2 and NFM wave-2 | 0.199** | 0.252** | 0.165** |
| AUDIT-C wave-2 and Positive parent-child relationship wave-2 | -0.103** | -0.057^a^ | -0.131** |
| FM wave-2 and NFM wave-2 | 0.265** | 0.293** | 0.245** |
| FM wave-2 and Positive parent-child relationship wave-2 | -0.324** | -0.182** | -0.416** |
| NFM wave-2 and Positive parent-child relationship wave-2 | -0.116** | -0.101** | -0.130** |
| **Wave-1 and Wave-2** | | | |
| AUDIT-C wave-1 and AUDIT-C wave-2 | 0.389** | 0.325** | 0.433** |
| FM wave-1 and FM wave-2 | 0.360** | 0.282** | 0.407** |
| NFM wave-1 and NFM wave-2 | 0.295** | 0.271** | 0.310** |

AUDIT-C: Alcohol Use Disorders Identification Test-Consumption; FM: Family maltreatment; NFM: maltreatment by a non-family member.

*r_s_=* Spearman’s correlation coefficient

**< 0.001

*< 0.05

^a^Not significant

#### S7 Table. Interaction effect of *MAOA*-uVNTR genotype, maltreatment and the positive dimensions of the parent-child relationship on alcohol consumption scores in females at wave-2.

1. *MAOA-*uVNTR genotype × FM × positive dimension of the parent-child relationship

| **Three-way interaction** | ***F*** | ***Δ R^2^* (%)** | ***b*** | ***p*** | ***LLCI*** | ***ULCI*** |
| --- | --- | --- | --- | --- | --- | --- |
| *MAOA*-uVNTR genotype × FM × Warmth^#^ | 6.442 | 0.7 | -0.135 | **0.011** | -0.240 | -0.031 |
| *MAOA*-uVNTR genotype × FM × Autonomy support^¤^ | 2.749 | 0.3 | -0.084 | 0.098 | -0.182 | 0.015 |
| *MAOA*-uVNTR genotype × FM × Structure^$^ | 3.442 | 0.4 | -0.097 | 0.064 | -0.199 | 0.006 |

1. *MAOA-*uVNTR genotype × NFM × positive dimension of the parent-child relationship

| **Three-way interaction** | ***F*** | ***Δ R^2^* (%)** | ***b*** | ***p*** | ***LLCI*** | ***ULCI*** |
| --- | --- | --- | --- | --- | --- | --- |
| *MAOA*-uVNTR genotype × NFM × Warmth^##^ | 8.980 | 1 | -0.354 | **0.003** | -0.586 | -0.122 |
| *MAOA*-uVNTR genotype × NFM × Autonomy support ^¤¤^ | 6.820 | 0.8 | -0.306 | **0.009** | -0.536 | -0.076 |
| *MAOA*-uVNTR genotype × NFM × Structure ^$$^ | 3.739 | 0.4 | -0.234 | 0.053 | -0.472 | 0.004 |

FM: Family maltreatment; NFM: Maltreatment by a non-family member.

Significant interaction effects are marked in **bold**. *b: beta-coefficient; F: F-test; FM: Family maltreatment; LLCI: Lower limit of confidence interval; Max: Maximum; Min: Minimum; se: standard error; NFM: maltreatment by a non-family member; t: t-statistics; ULCI: Upper limit of confidence interval.*

^#^Model: *R^2^* = 0.051, *F* (7,840) = 6.613, *p* < 0.001;

^¤^Model: *R^2^* = 0.055, *F* (7,840) = 7.013, *p* < 0.001; ^$^Model: *R^2^* = 0.045, *F* (7,840) = 5.664, *p* < 0.001;

^##^Model: *R^2^* = 0.060, *F* (7,840) = 7.630, *p* < 0.001; ^¤¤^Model: *R^2^* = 0.054, *F* (7,840) = 6.873, *p* < 0.001; ^$$^Model: *R^2^* = 0.069, *F* (7,840) = 8.900, *p* < 0.001.

#### S8a Table. Effect of all covariates on the model of AUDIT-C score (Wave-2) containing three-way interaction between *MAOA*-uVNTR genotype × family maltreatment × positive parent-child relationship in females.

| Model (R^2^ = 0.376, Adjusted R^2^= 0.349) | *F* | *p* value |
| --- | --- | --- |
| Intercept | 0.000 | 0.985 |
| *MAOA*-uVNTR | 0.019 | 0.890 |
| FM | 0.393 | 0.531 |
| PPCR | 0.117 | 0.733 |
| Behavioural problems 1^a^ | 2.179 | 0.140 |
| Behavioural problems 2 ^a^ | 4.874 | **0.028** |
| Other negative environmental factors^a^ | 4.535 | **0.034** |
| Parent’s alcohol use and Positive life events | 1.903 | 0.168 |
| *MAOA*-uVNTR x FM | 0.799 | 0.372 |
| *MAOA*-uVNTR x Behavioural problems 1 ^a^ | 2.736 | 0.099 |
| *MAOA*-uVNTR x Behavioural problems 2^a^ | 0.346 | 0.556 |
| *MAOA*-uVNTR x Other negative environmental factors^a^ | 2.817 | 0.094 |
| *MAOA*-uVNTR x Parent’s alcohol use and Positive life events | 0.321 | 0.571 |
| FM x PPCR | 0.296 | 0.587 |
| FM x Behavioural problems 1^a^ | 0.041 | 0.839 |
| FM x Behavioural problems 2^a^ | 2.718 | 0.100 |
| FM x Other negative environmental factors ^a^ | 2.690 | 0.101 |
| FM x Parent’s alcohol use and Positive life events | 1.322 | 0.251 |
| *MAOA*-uVNTR x PPCR | 0.022 | 0.883 |
| PPCR x Behavioural problems 1^a^ | 1.257 | 0.263 |
| PPCR x Behavioural problems 2^a^ | 0.569 | 0.451 |
| PPCR x Other negative environmental factors^a^ | 4.732 | **0.030** |
| PPCR x Parent’s alcohol use and Positive life events | 1.389 | 0.239 |
| *MAOA*-uVNTR x FM x Behavioural problems 1^a^ | 2.631 | 0.105 |
| *MAOA*-uVNTR x FM x Behavioural problems 2^a^ | 2.299 | 0.130 |
| *MAOA*-uVNTR x FM x Other negative environmental factors^a^ | 0.153 | 0.696 |
| *MAOA*-uVNTR x FM x Parent’s alcohol use and Positive life events | 0.578 | 0.447 |
| *MAOA*-uVNTR x PPCR x Behavioural problems 1^a^ | 2.414 | 0.121 |
| *MAOA*-uVNTR x PPCR x Behavioural problems 2^a^ | 0.061 | 0.805 |
| *MAOA*-uVNTR x PPCR x Other negative environmental factors^a^ | 3.250 | 0.072 |
| *MAOA*-uVNTR x PPCR x Parent’s alcohol use and Positive life events | 0.490 | 0.484 |
| FM x PPCR x Behavioural problems 1^a^ | 0.150 | 0.699 |
| FM x PPCR x Behavioural problems 2^a^ | 3.247 | 0.072 |
| FM x PPCR x Other negative environmental factors^a^ | 1.896 | 0.169 |
| *MAOA*-uVNTR x FM x PPCR | 0.639 | 0.424 |

FM: family maltreatment; PPCR: positive parent-child relationship.

Significant *p*-values are marked in **BOLD**

^a^See S2 Table for the list for covariates included in this composite variable.

#### Table S8b Effect of each covariate on the model of AUDIT-C score (Wave-2) containing three-way interaction between *MAOA*-uVNTR genotype × family maltreatment × positive parent-child relationship in females.

|  | **Univariate GLM Model summary** | | | ***MAOA*-uVNTR genotype × family maltreatment × positive parent-child relationship** | | **Parameter estimates for *MAOA*-SL/LL × family maltreatment × positive parent-child**  **relationship** | | | | | |
| --- | --- | --- | --- | --- | --- | --- | --- | --- | --- | --- | --- |
|  | ***n*** | ***R^2^*** | ***Adjusterd R^2^*** | ***F (df=1)*** | ***p*** | ***b*** | **SE** | ***t*** | ***P*** | ***LLCI*** | ***ULCI*** |
| Unadjusted model | 848 | 0.052 | 0.044 | 4.703 | **0.030** | -0.043 | 0.020 | -2.169 | **0.030** | -0.083 | -0.004 |
| **Covariates- Environmental factors** |  |  |  |  |  |  |  |  |  |  |  |
| AUDIT-C parent | 841 | 0.096 | 0.081 | 5.686 | **0.017** | -0.050 | 0.021 | -2.385 | **0.017** | -0.092 | -0.009 |
| Family maltreatment, Wave-1 | 835 | 0.070 | 0.054 | 0.758 | 0.384 | -0.022 | 0.026 | -0.871 | 0.384 | -0.073 | 0.028 |
| Non-family maltreatment, Wave-1 | 835 | 0.065 | 0.049 | 0.598 | 0.440 | -0.019 | 0.024 | -0.773 | 0.440 | -0.066 | 0.029 |
| Non-family maltreatment, Wave-2 | 848 | 0.80 | 0.065 | 1.285 | 0.257 | -0.025 | 0.022 | -1.113 | 0.257 | -0.068 | 0.018 |
| Negative parent-child relationship | 848 | 0.070 | 0.055 | 2.092 | 0.148 | -0.053 | 0.036 | -1.446 | 0.148 | -0.124 | 0.019 |
| Bullied in school, Wave-1 | 835 | 0.064 | 0.048 | 3.347 | 0.068 | -0.041 | 0.022 | -1.830 | 0.068 | -0.084 | 0.003 |
| Bullied in school, Wave-2 | 845 | **0.056** | **0.040** | 3.922 | **0.048** | -0.045 | 0.023 | -1.980 | **0.048** | -0.090 | 0.000 |
| Positive life events | 841 | 0.061 | 0.045 | 3.280 | 0.071 | -0.038 | 0.021 | -1.811 | 0.071 | -0.079 | 0.003 |
| Negative life events | 841 | 0.076 | 0.061 | 3.244 | 0.072 | -0.051 | 0.028 | -1.801 | 0.072 | -0.106 | 0.005 |
| **Covariates- Behavioural factors** |  |  |  |  |  |  |  |  |  |  |  |
| AUDIT-C, Wave-1 | 838 | 0.210 | 0.196 | 3.133 | 0.007 | -0.037 | 0.021 | -1.770 | 0.077 | -0.077 | 0.004 |
| Nicotine use, Wave-1 | 848 | 0.129 | 0.114 | 3.555 | 0.060 | -0.039 | 0.021 | -1.886 | 0.060 | -0.080 | 0.002 |
| Nicotine use, Wave-2 | 848 | 0.324 | 0.312 | 2.745 | 0.098 | -0.033 | 0.020 | -1.657 | 0.098 | -0.072 | 0.003 |
| Use of illicit substances, Wave-1 | 843 | 0.069 | 0.054 | 2.924 | 0.088 | -0.035 | 0.021 | -1.710 | 0.088 | -0.076 | 0.005 |
| Violent Delinquency, Wave-1 | 847 | 0.073 | 0.057 | 2.087 | 0.149 | -0.031 | 0.022 | -1.445 | 0.149 | -0.074 | 0.011 |
| Non-violent Delinquency, Wave-1 | 847 | 0.186 | 0.172 | 4.202 | **0.041** | -0.043 | 0.021 | -2.050 | **0.041** | -0.084 | -0.002 |
| Violent Delinquency, Wave-2 | 848 | 0.065 | 0.049 | 1.791 | 0.181 | -0.029 | 0.022 | -1.338 | 0.181 | -0.072 | 0.014 |
| Non-violent Delinquency, Wave-2 | 848 | 0.175 | 0.161 | 0.667 | 0.414 | -0.017 | 0.021 | -0.817 | 0.414 | -0.058 | 0.024 |

Significant *p*-values for the three-way interaction after adjustment are marked in **BOLD.**

#### S9 Table. Description of the model showing three-way interaction effect of *MAOA*-uVNTR genotype, FM, and positive parent-child relationship on alcohol consumption scores at wave-2 in males.

| Model | *b* | | *se* | *t* | *p* | *LLCI* | *ULCI* |
| --- | --- | --- | --- | --- | --- | --- | --- |
| Constant |  | 5.270 | 1.164 | 4.527 | 0.000 | 2.983 | 7.557 |
| FM | *b_1_* | -0.270 | 0.496 | -0.545 | 0.586 | -1.244 | 0.704 |
| *MAOA*-uVNTR genotype | *b_2_* | -0.082 | 2.091 | -0.039 | 0.969 | -4.189 | 4.025 |
| Positive parent-child relationship | *b_3_* | -0.068 | 0.041 | -1.675 | 0.094 | -0.148 | 0.012 |
| *MAOA*-uVNTR genotype × FM | *b_4_* | -0.199 | 0.645 | -0.308 | 0.758 | -1.465 | 1.068 |
| FM × Positive parent-child relationship | *b_5_* | 0.012 | 0.019 | 0.595 | 0.552 | -0.027 | 0.050 |
| *MAOA*-uVNTR genotype × Positive parent-child relationship | *b_6_* | 0.004 | 0.072 | 0.059 | 0.953 | -0.138 | 0.146 |
| ***MAOA*-uVNTR genotype × FM ×**  **Positive parent-child relationship** | ***b_7_*** | 0.003 | 0.025 | 0.124 | 0.901 | -0.047 | 0.053 |

Model: (*R^2^* = 0.009, *F* (7,560) = 6.613, *p* = 0.641).

Change in R^2^ due to three-way interaction: *F* (1, 560) = 0.015, Δ*R^2^* = 0.000, *p* = 0.901)

*b: beta-coefficient; FM: Family maltreatment; LLCI: Lower limit of confidence interval; Max: Maximum; Min: Minimum; se: standard error; t: t-statistics; ULCI: Upper limit of confidence interval.*

#### S10a Table. Effect of all covariates on the model of AUDIT-C score (Wave-2) containing three-way interaction between *MAOA*-uVNTR genotype × non-family maltreatment × positive parent-child relationship in females.

| Model (R^2^ = 0.376, Adjusted R^2^= 0.349) | *F* | *p* value |
| --- | --- | --- |
| Intercept | 0.012 | 0.912 |
| *MAOA*-uVNTR | 0.023 | 0.879 |
| NFM | 0.003 | 0.955 |
| PPCR | 0.071 | 0.790 |
| Behavioural problems 1^a^ | 4.602 | **0.032** |
| Behavioural problems 2 ^a^ | 2.359 | 0.125 |
| Other negative environmental factors^a^ | 1.501 | 0.221 |
| Parent’s alcohol use and Positive life events | 1.079 | 0.299 |
| *MAOA*-uVNTR x NFM | 0.055 | 0.814 |
| *MAOA*-uVNTR x Behavioural problems 1 ^a^ | 2.162 | 0.142 |
| *MAOA*-uVNTR x Behavioural problems 2^a^ | 0.271 | 0.603 |
| *MAOA*-uVNTR x Other negative environmental factors^a^ | 2.807 | 0.094 |
| *MAOA*-uVNTR x Parent’s alcohol use and Positive life events | 0.999 | 0.318 |
| NFM x PPCR | 0.404 | 0.525 |
| NFM x Behavioural problems 1^a^ | 1.780 | 0.183 |
| NFM x Behavioural problems 2^a^ | 0.423 | 0.516 |
| NFM x Other negative environmental factors ^a^ | 0.067 | 0.796 |
| NFM x Parent’s alcohol use and Positive life events | 0.939 | 0.333 |
| *MAOA*-uVNTR x PPCR | 0.021 | 0.885 |
| PPCR x Behavioural problems 1^a^ | 3.567 | 0.059 |
| PPCR x Behavioural problems 2^a^ | 0.032 | 0.857 |
| PPCR x Other negative environmental factors^a^ | 1.939 | 0.164 |
| PPCR x Parent’s alcohol use and Positive life events | 0.659 | 0.417 |
| *MAOA*-uVNTR x NFM x Behavioural problems 1^a^ | 0.797 | 0.372 |
| *MAOA*-uVNTR x NFM x Behavioural problems 2^a^ | 2.186 | 0.140 |
| *MAOA*-uVNTR x NFM x Other negative environmental factors^a^ | 0.252 | 0.616 |
| *MAOA*-uVNTR x NFM x Parent’s alcohol use and Positive life events | 0.055 | 0.814 |
| *MAOA*-uVNTR x PPCR x Behavioural problems 1^a^ | 2.304 | 0.129 |
| *MAOA*-uVNTR x PPCR x Behavioural problems 2^a^ | 0.043 | 0.836 |
| *MAOA*-uVNTR x PPCR x Other negative environmental factors^a^ | 3.313 | 0.069 |
| *MAOA*-uVNTR x PPCR x Parent’s alcohol use and Positive life events | 1.282 | 0.258 |
| NFM x PPCR x Behavioural problems 1^a^ | 1.096 | 0.295 |
| NFM x PPCR x Behavioural problems 2^a^ | 0.511 | 0.475 |
| NFM x PPCR x Other negative environmental factors^a^ | 0.680 | 0.410 |
| *MAOA*-uVNTR x NFM x PPCR | 0.249 | 0.618 |

NFM: non-family maltreatment; PPCR: positive parent-child relationship.

Significant *p*-values are marked in **BOLD**

^a^See S2 Table for the list for covariates included in this composite variable.

|  | **Univariate GLM Model summary** | | | ***MAOA*-uVNTR genotype × non**- **family maltreatment × positive parent-child relationship** | | **Parameter estimates for *MAOA*-SL/LL × non-family maltreatment × positive parent-child**  **relationship** | | | | | |
| --- | --- | --- | --- | --- | --- | --- | --- | --- | --- | --- | --- |
|  | ***n*** | ***R^2^*** | ***Adjusterd R^2^*** | ***F (df=1)*** | ***p*** | ***b*** | **SE** | ***t*** | ***P*** | ***LLCI*** | ***ULCI*** |
| Unadjusted model | 848 | 0.068 | 0.060 | 8.977 | **0.003** | *-0.131* | 0.011 | 0.849 | **-0.131** | 0.044 | -2.996 |
| **Covariates- Environmental factors** |  |  |  |  |  |  |  |  |  |  |  |
| AUDIT-C parent | 841 | 0.105 | 0.089 | 1.596 | 0.207 | -0.075 | 0.060 | -1.263 | 0.207 | -0.193 | 0.042 |
| Family maltreatment, Wave-1 | 835 | **0.081** | **0.065** | **4.500** | **0.034** | **-0.111** | **0.052** | **-2.121** | **0.034** | **-0.214** | **-0.008** |
| Non-family maltreatment, Wave-1 | 835 | **0.086** | **0.070** | **6.090** | **0.014** | **-0.112** | **0.045** | **-2.468** | **0.014** | **-0.201** | **-0.023** |
| Non-family maltreatment, Wave-2 | 848 | **0.80** | **0.065** | **5.974** | **0.015** | **-0.145** | **0.059** | **-2.444** | **0.015** | **-0.262** | **-0.029** |
| Negative parent-child relationship | 848 | 0.088 | 0.072 | 0.896 | 0.344 | -0.068 | 0.072 | -0.947 | 0.344 | -0.210 | 0.073 |
| Bullied in school, Wave-1 | 835 | **0.077** | **0.061** | **4.860** | **0.028** | **-0.118** | **0.053** | **-2.205** | **0.028** | **-0.223** | **-0.013** |
| Bullied in school, Wave-2 | 845 | **0.073** | **0.058** | **6.812** | **0.009** | **-0.119** | **0.046** | **-2.610** | **0.009** | **-0.209** | **-0.030** |
| Positive life events | 841 | **0.076** | **0.060** | **9.007** | **0.003** | **-0.139** | **0.046** | **-3.001** | **0.003** | **-0.230** | **-0.048** |
| Negative life events | 841 | **0.091** | **0.076** | **7.510** | **0.006** | **-0.135** | **0.049** | **-2.740** | **0.006** | **-0.232** | **-0.038** |
| **Covariates- Behavioural factors** |  |  |  |  |  |  |  |  |  |  |  |
| AUDIT-C, Wave-1 | 838 | 0.208 | **0.195** | 9.656 | **0.002** | -0.134 | 0.043 | -3.107 | **0.002** | **-0.219** | **-0.049** |
| Nicotine use, Wave-1 | 848 | **0.136** | **0.122** | **9.409** | **0.002** | **-0.136** | **0.044** | **-3.067** | **0.002** | **-0.222** | **-0.049** |
| Nicotine use, Wave-2 | 848 | 0.326 | 0.315 | 2.256 | 0.134 | -0.059 | 0.039 | -1.502 | 0.134 | -0.137 | 0.018 |
| Use of illicit substances, Wave-1 | 843 | **0.078** | **0.063** | **7.582** | **0.006** | **-0.122** | **0.044** | **-2.754** | **0.006** | **-0.208** | **-0.035** |
| Violent Delinquency, Wave-1 | 847 | **0.086** | **0.070** | **5.714** | **0.017** | **-0.107** | **0.045** | **-2.390** | **0.017** | **-0.195** | **-0.019** |
| Non-violent Delinquency, Wave-1 | 847 | **0.186** | **0.172** | **11.128** | **< 0.001** | **-0.155** | **0.046** | **-3.336** | **< 0.001** | **-0.246** | **-0.064** |
| Violent Delinquency, Wave-2 | 848 | **0.078** | **0.062** | **6.139** | 0.013 | **-0.116** | **0.047** | **-2.478** | **0.013** | **-0.208** | **-0.024** |
| Non-violent Delinquency, Wave-2 | 848 | **0.168** | **0.154** | **3.849** | **0.050** | **-0.090** | **0.046** | **-1.962** | **0.050** | **-0.181** | **4.599e^-5^** |

#### S10b Table. Effect of each covariate on the model of AUDIT-C score (Wave-2) containing three-way interaction between *MAOA*-uVNTR genotype × non-family maltreatment × positive parent-child relationship in females.

Significant *p*-values for the three-way interaction after adjustment are marked in **BOLD.**

#### S11 Table. Description of the model showing three-way interaction effect of *MAOA*-uVNTR genotype, NFM, and positive parent-child relationship on alcohol consumption scores at wave-2 in males.

| Model | *b* | | *se* | *t* | *p* | *LLCI* | *ULCI* |
| --- | --- | --- | --- | --- | --- | --- | --- |
| Constant |  | 4.766 | 1.016 | 4.690 | 0.000 | 2.770 | 6.762 |
| NFM | *b_1_* | -0.115 | 0.957 | -0.120 | 0.904 | -1.995 | 1.765 |
| *MAOA*-uVNTR genotype | *b_2_* | -1.214 | 1.755 | -0.692 | 0.489 | -4.661 | 2.233 |
| Positive parent-child relationship | *b_3_* | -0.059 | 0.035 | -1.661 | 0.097 | -0.129 | 0.011 |
| *MAOA*-uVNTR genotype × NFM | *b_4_* | -0.695 | 1.438 | -0.483 | 0.629 | -3.519 | 2.130 |
| NFM × Positive parent-child  Relationship | *b_5_* | 0.032 | 0.035 | 0.909 | 0.364 | -0.037 | 0.100 |
| *MAOA*-uVNTR genotype × Positive parent-child relationship | *b_6_* | 0.028 | 0.061 | 0.459 | 0.646 | -0.092 | 0.148 |
| ***MAOA*-uVNTR genotype × NFM ×**  **Positive parent-child relationship** | ***b_7_*** | 0.044 | 0.052 | 0.850 | 0.396 | -0.058 | 0.146 |

Model: (*R^2^* = 0.098, *F* (7,559) = 8.711, *p* = 0.000).

Change in R^2^ due to three-way interaction: *F* (1, 559) = 0.722, Δ*R^2^* = 0.001, *p* = 0.396)

*b: beta-coefficient; FM: Family maltreatment; LLCI: Lower limit of confidence interval; Max: Maximum; Min: Minimum; se: standard error; t: t-statistics; ULCI: Upper limit of confidence interval.*


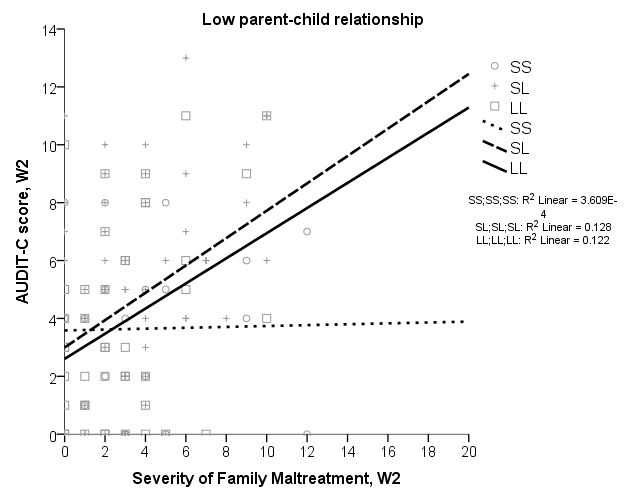


(A)


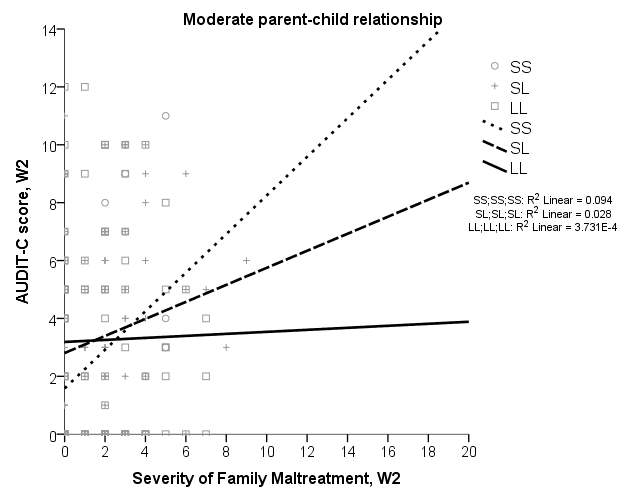


(B)


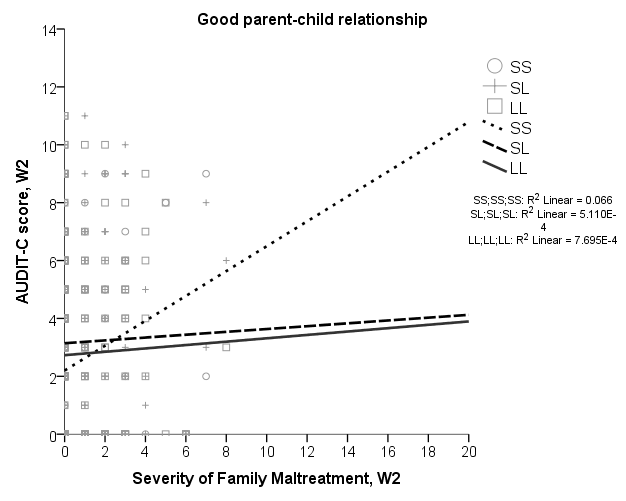


(C)

#### S1 Fig. Graphical illustration of the interaction between three variants of *MAOA* genotype and family maltreatment on alcohol consumption in adolescent females with poor (A), average (B), and good (C) positive parent-child relationship.


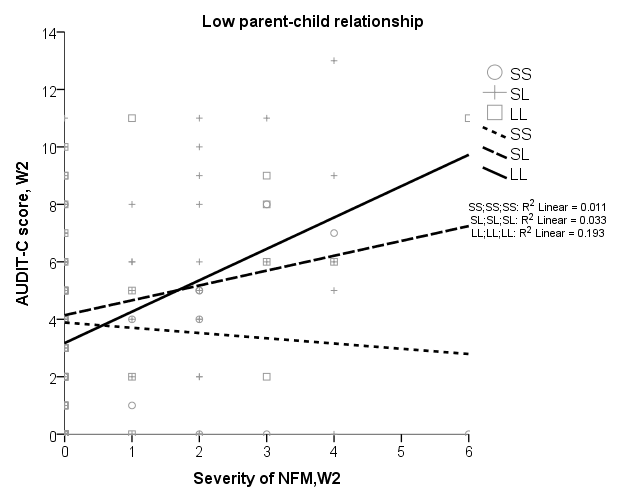


(A)

(B)


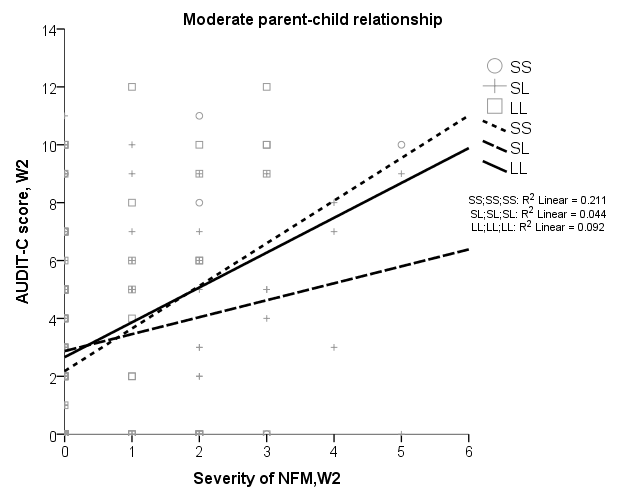


(C)


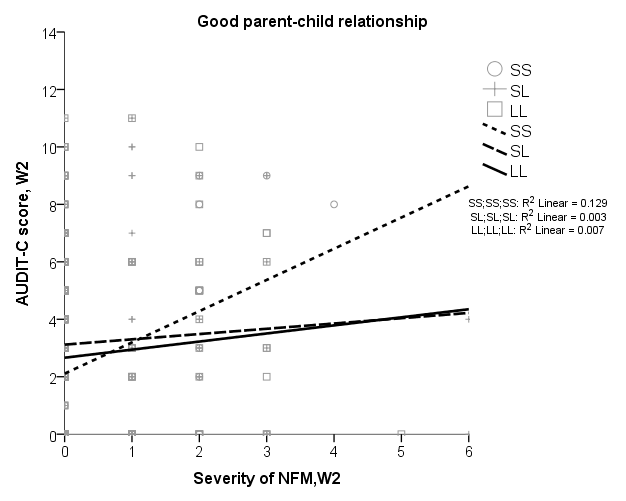


#### S2 Fig. Graphical illustration of the interaction between three variants of *MAOA* genotype and maltreatment by a non-familial member (NFM) on alcohol consumption among adolescent females with poor (A), average (B), and good (C) positive parent-child relationship.

**
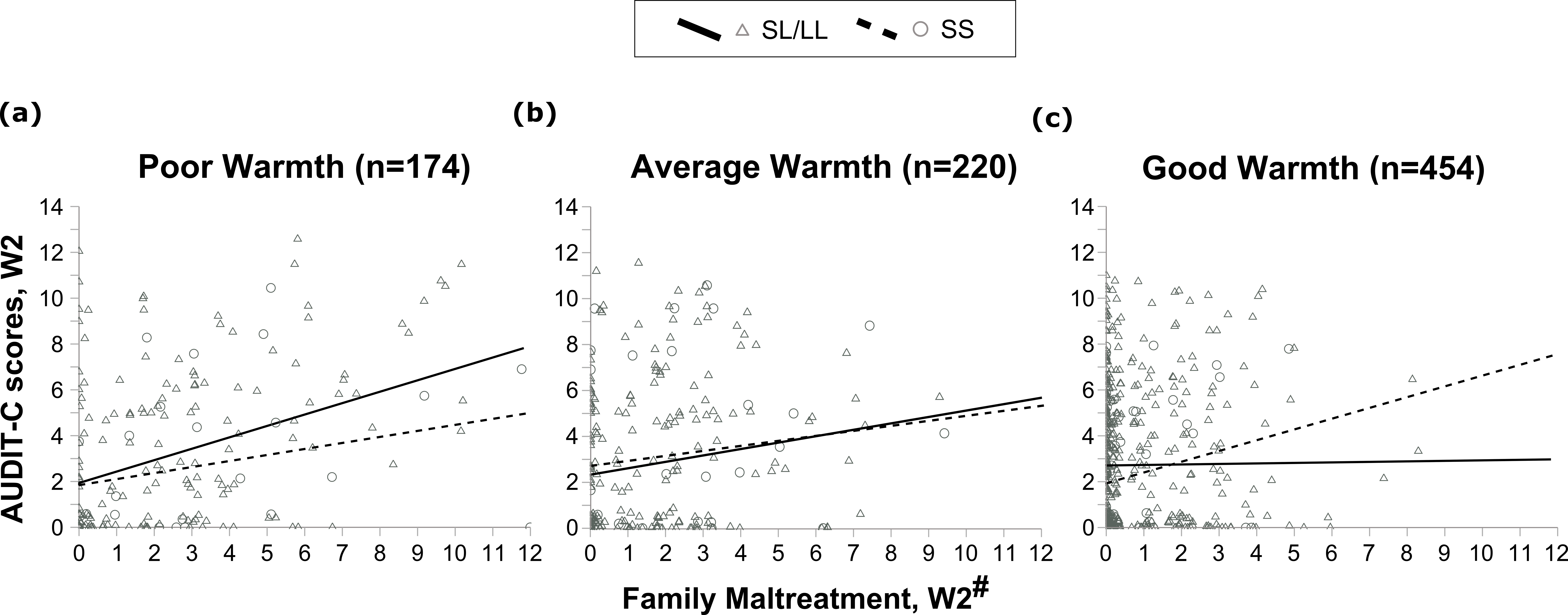
**

#### **S3 Fig.** Scatter plots showing association between alcohol consumption and family maltreatment in *MAOA*-SL/LL and *MAOA*-SS allele carrying females having a poor, average, and good warmth. (a) poor warmth (*MAOA*-SL/LL carriers: *n* = 150, *R^2^* = 0.144, *r_s_* = 0.325, *p* = <0.001, slope = 0.5; *MAOA*-SS carriers: *n* = 24, *R^2^* = 0.072, *r_s_* = 0.328, *p* = 0.117, slope = 0.26). (b) average warmth (*MAOA*-SL/LL carriers: *n* = 191, *R^2^* = 0.026, *r_s_* = 0.187, *p* = 0.009, slope = 0.28; *MAOA*-SS carriers: *n* = 29, *R^2^* = 0.020, *r_s_* = 0.205, *p* = 0.287, slope = 0.22). (c) good warmth (*MAOA*-SL/LL carriers: *n* = 404, *R^2^* = 9.891e^-5^, *r_s_* = 0.007, *p* = 0.885, slope = 0.02; *MAOA*-SS carriers: *n* = 50, *R^2^* = 0.046, *r_s_* = 0.166, *p* = 0.248, slope = 0.47). ^#^ Here the family maltreatment score is shown from 0-12, as 12 was the highest score in the data but the total score ranges from 0-20.

**
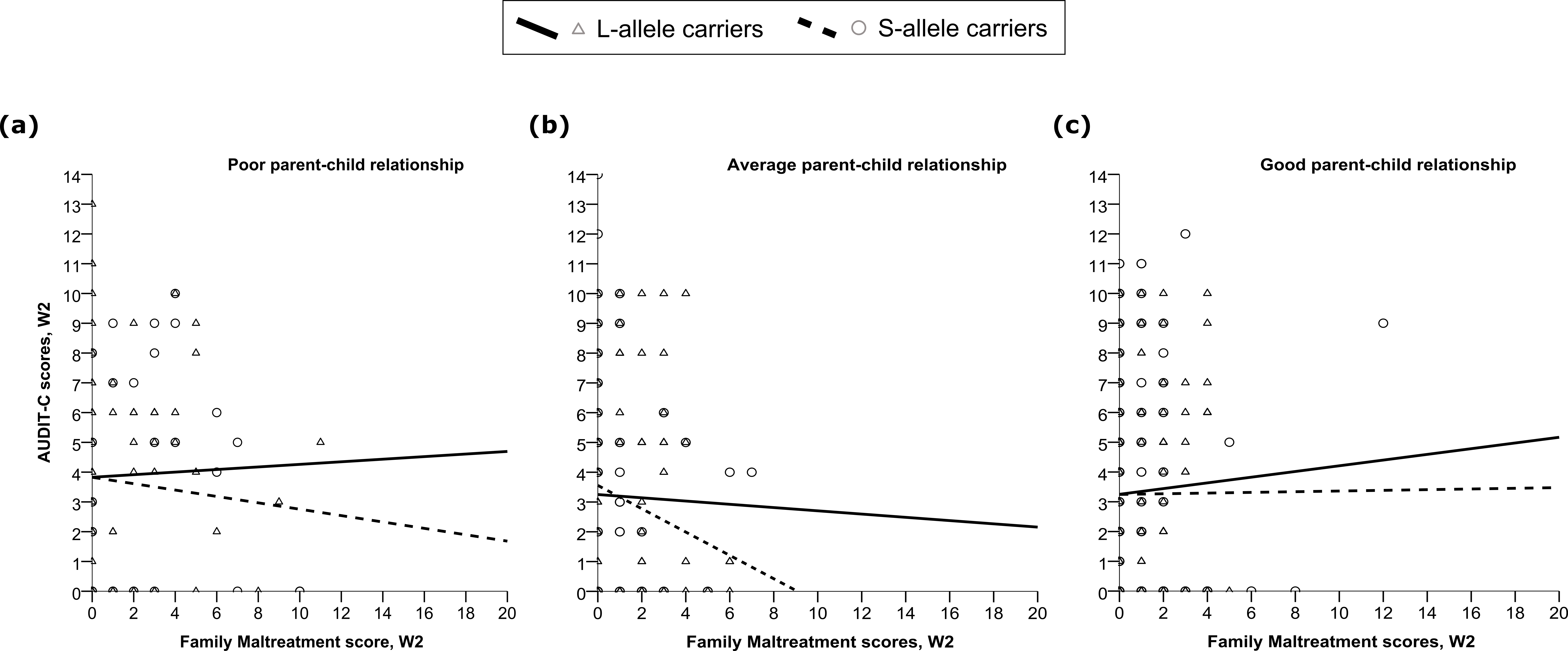
**

#### S4 Fig. Scatter plots showing no association between alcohol consumption and family maltreatment in *MAOA*-S and *MAOA*-L allele carrying males having (a) poor parent-child relationship, (b) average parent-child relationship, and (c) good parent-child relationship.


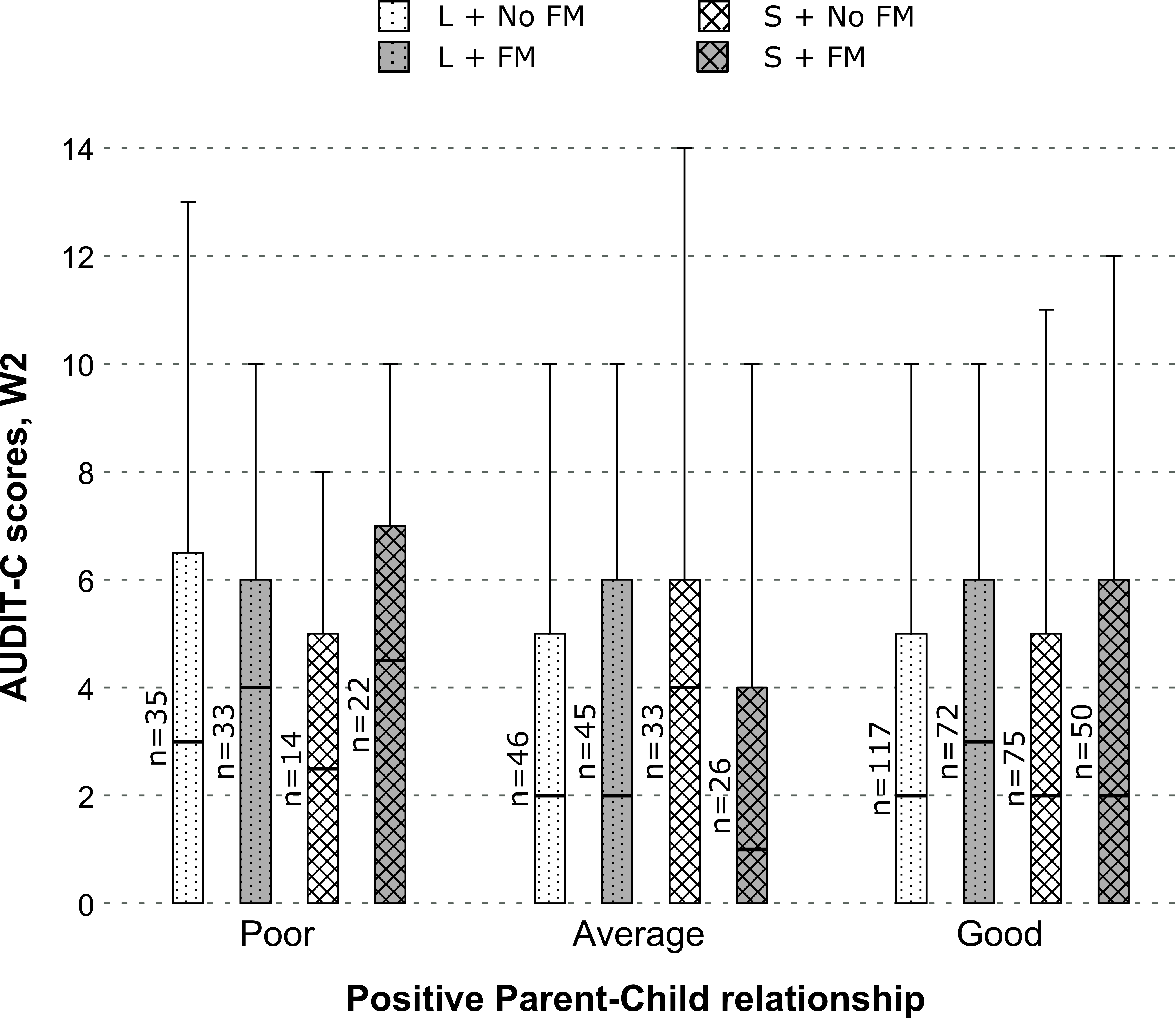


#### S5 Fig. Box plots showing pairwise comparisons to unpack the three-way interaction effect of *MAOA*-uVNTR genotype × family maltreatment × positive parent-child relationship on alcohol consumption in the males. None of the pairwise comparisons were significantly different (*H* (11) = 6.477, *p* = 0.840).





#### **S6 Fig.** Scatter plots showing association between alcohol consumption and non-family maltreatment in MAOA*-SL/LL* and MAOA*-SS* allele carrying females depending on the three dimensions of the positive parent-child relationship.

(a) Poor warmth (*MAOA*-SL/LL carriers: *n* = 150, *R^2^* = 0.105, ***r_s_* = 0.244, *p* = 0.003**, **slope = 0.95**; *MAOA*-SS carriers: *n* = 24, *R^2^* = 0.014, *r_s_* = 0.179, *p* = 0.404**, slope = 0.25**)*.* (b) Average warmth (*MAOA*-SL/LL carriers: *n* = 191, *R^2^* = 0.036, ***r_s_* = 0.187, *p* = 0.010**, **slope = 0.56**;

*MAOA*-SS carriers: *n* = 29, *R^2^* = 0.101, *r_s_* = 0.219, *p* = 0.253, slope = 0.9). (c) Good warmth **(***MAOA*-SL/LL carriers: *n* = 404, *R^2^* = 0.008, *r_s_* = 0.086, *p* = 0.085, slope = 0.32; *MAOA*-SS carriers: *n* = 50, *R^2^* = 0.156, ***r_s_* = 0.357, *p* = 0.011**, slope = 1.31). (d) Poor structure (*MAOA*-SL/LL carriers: *n* = 120, *R^2^* = 0.113, ***r_s_* = 0.295, *p* = 0.001**, slope = 0.98; *MAOA*-SS carriers: *n* = 15, *R^2^* = 0.010, *r_s_* = -0.043, *p* = 0.880, slope = -0.16). (e) Average structure (*MAOA*-SL/LL carriers: *n* = 310, *R^2^* = 0.023, ***r_s_* = 0.126, *p* = 0.027**, slope = 0.46; *MAOA*-SS carriers: *n* = 40, *R^2^* = 0.178, ***r_s_* = 0.373, *p* = 0.018**, slope = 1.14). (f) Good structure (*MAOA*-SL/LL carriers: *n* = 315, *R^2^* = 0.009, *r_s_* = 0.100, *p* = 0.077, slope = 0.34; *MAOA*-SS carriers: *n* = 48, *R^2^* = 0.042, *r_s_* = 0.168, *p* = 0.253, slope = 0.72). (g) Poor autonomy **(***MAOA*-SL/LL carriers: *n* = 160, *R^2^* = 0.056, ***r_s_* = 0.200, *p* = 0.011**; slope = 0.71; *MAOA*-SS carriers: *n* = 29, *R^2^* = 0.030, *r_s_* = 0.137, *p* = 0.478, slope = 0.33). (h) Average autonomy (*MAOA*-SL/LL carriers: *n* = 228, *R^2^* = 0.030, *r_s_* = 0.106, *p* = 0.109, slope = 0.52; *MAOA*-SS carriers: *n* = 28, *R^2^* = 0.078, *r_s_* = 0.252, *p* = 0.196, slope = 0.89), and (i) Good autonomy **(***MAOA*-SL/LL carriers: *n* = 357, *R^2^* = 0.022, ***r_s_* = 0.151, *p* = 0.004,** slope = 0.51; *MAOA*-SS carriers: *n* = 46, *R^2^* = 0.153, *r_s_* = **0.303, *p* = 0.040,** slope = 1.39).


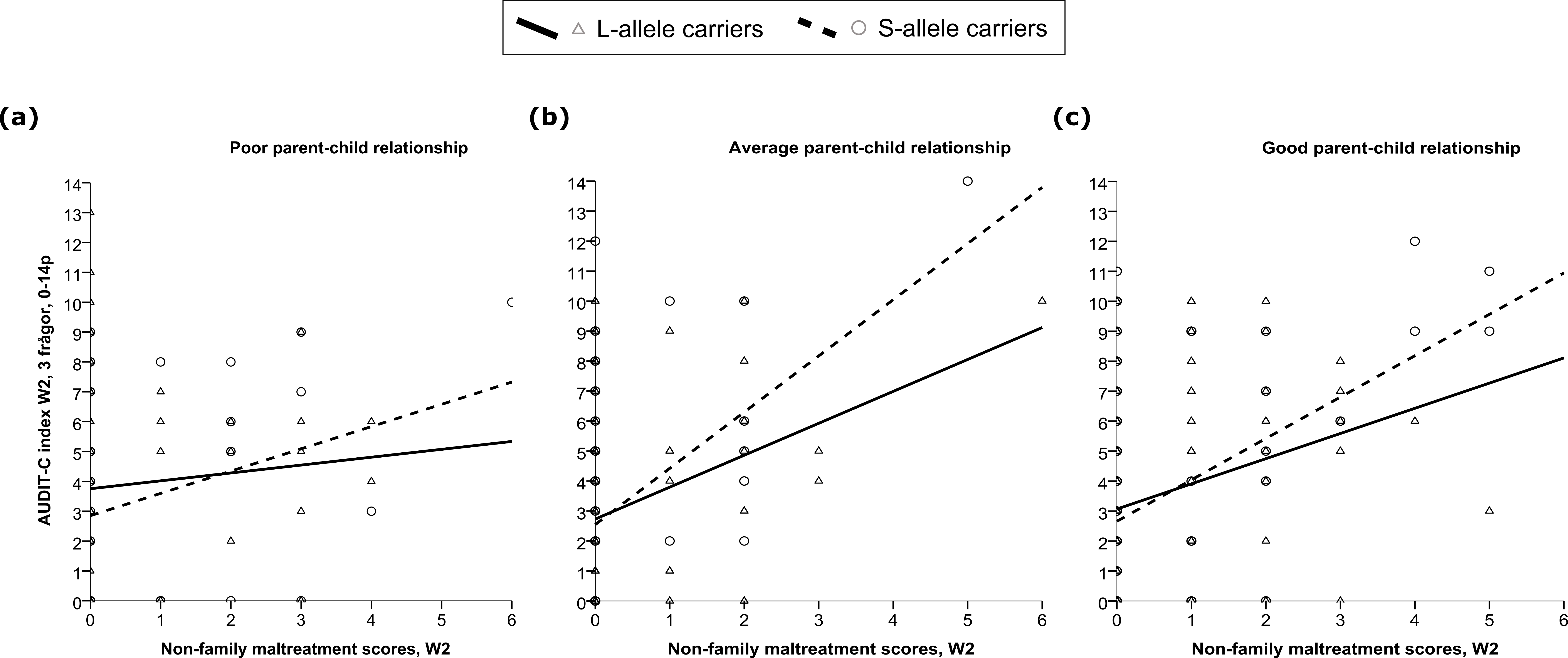


#### S7 Fig. Scatter plots showing no association between alcohol consumption and non-family maltreatment in *MAOA*-S and *MAOA*-L allele carrying males having (a) poor parent-child relationship, (b) average parent-child relationship, and (c) good parent-child relationship.


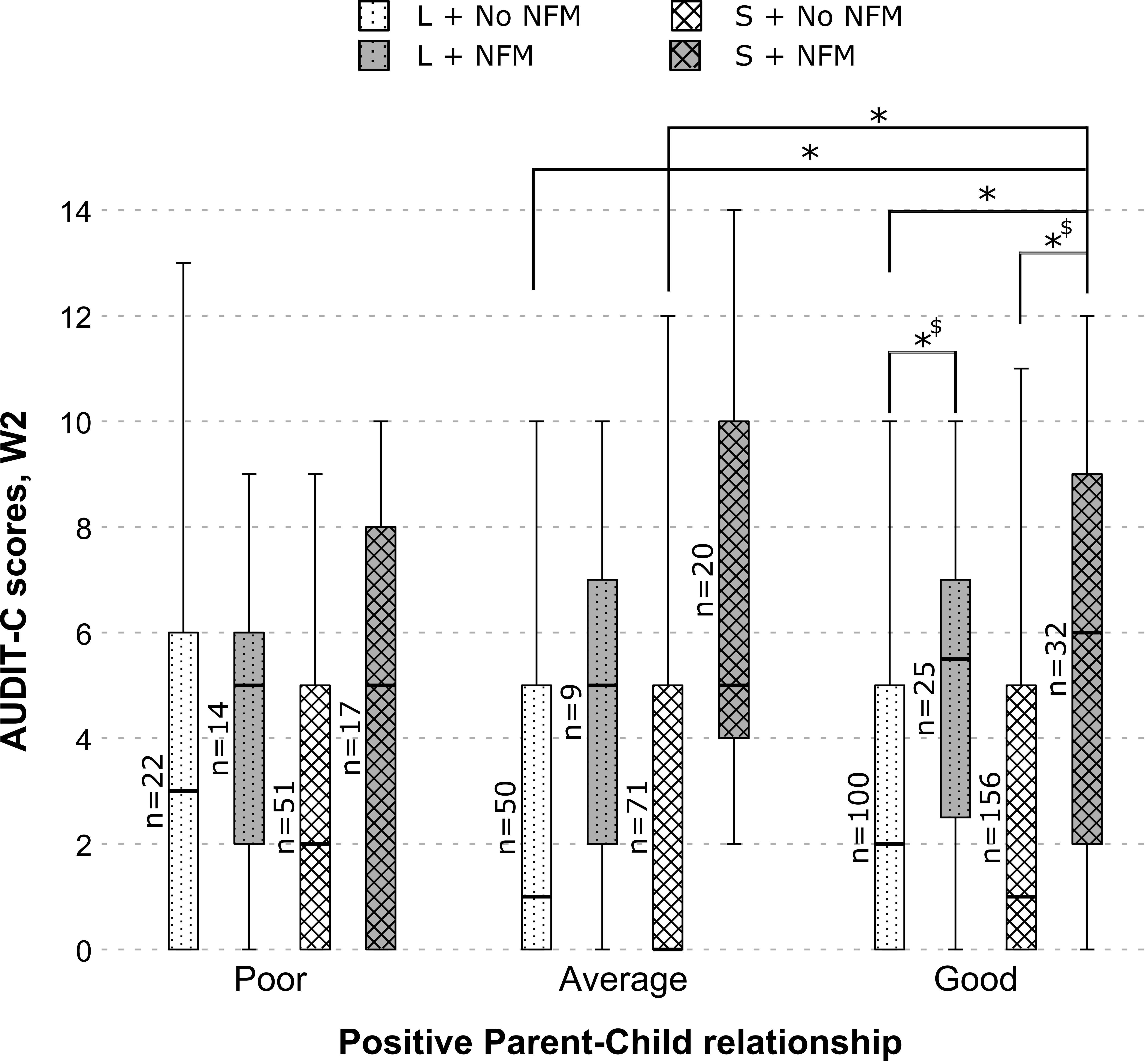


#### **S8 Fig.** Box plots showing pairwise comparisons to unpack the three-way interaction effect of *MAOA*-uVNTR genotype × non-family maltreatment × positive parent-child relationship on alcohol consumption in the males. The overall KW test revealed that the AUDIT-C scores differed between the groups (*H* (11) = 39.334, *p* < 0.001), but the pairwise comparisons showed that the two comparisons were mainly due to exposure to NFM (comparisons marked with “$”) and not due to quality of positive parent-child relationship, while rest were meaningless comparisons. Thus, there was no significant effect of the three-way interaction effect of *MAOA*-uVNTR genotype × non-family maltreatment × positive parent-child relationship on alcohol consumption in the males.

*Significant differences.

^$^ Effect of exposure to NFM: Irrespective of the good parent-child relationship, males carrying *MAOA*-S or *MAOA*-L allele exposed to NFM had higher AUDIT-C scores compared to their counterparts without exposure to NFM.


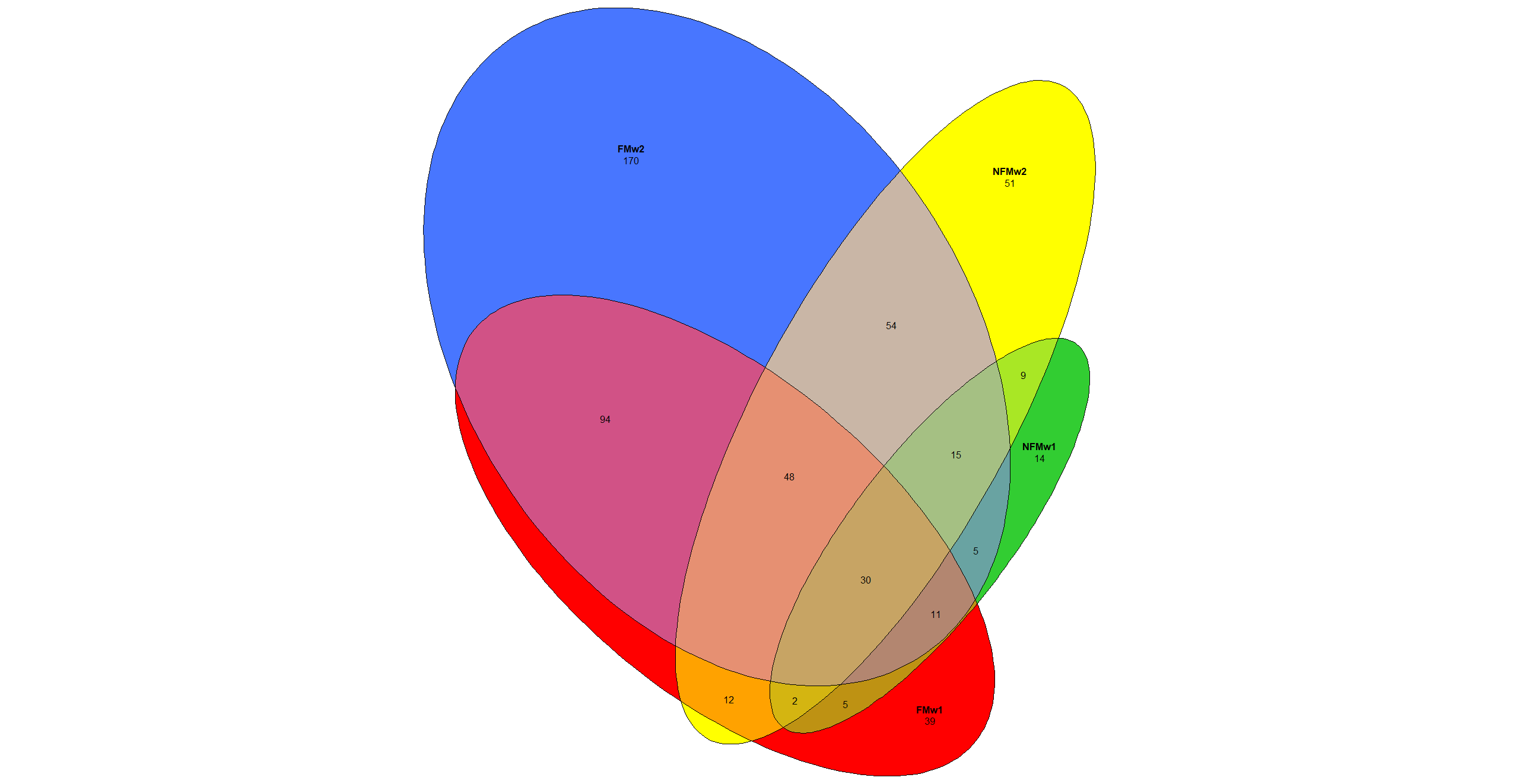


#### S9 Fig. Overlap of FM and NFM at wave-1 and -2 in females. The size of the circles is proportional to the number of participants who experienced a respective type of maltreatment. The amount of overlap between the circles is proportional to the co-occurrence of maltreatment types.

Among females who experienced FM at wave-1, 19.9 % also experienced NFM and, 75.9% experienced FM at wave-2, and 38.2% experienced NFM at wave-2.

FMw1: Family maltreatment at wave-1; FMw2: Family maltreatment at wave-2; NFMw1: maltreatment by a non-family member at wave-1; NFMw2: maltreatment by a non-family member at wave-2. The numbers indicate frequencies.

#### References

1. Skinner E, Johnson S, Snyder T. Six Dimensions of Parenting: A Motivational Model. Parenting. 2005;5(2):175-235.

2. Andershed H, Gustafson SB, Kerr M, Stattin H. The usefulness of self-reported psychopathy-like traits in the study of antisocial behaviour among non-referred adolescents. European Journal of Personality. 2002;16(5):383-402.

3. Allen JL, Rapee RM, Sandberg S. Assessment of Maternally Reported Life Events in Children and Adolescents: A Comparison of Interview and Checklist Methods. Journal of Psychopathology and Behavioral Assessment. 2012;34(2):204-15.

4. Nilsson KW, Comasco E, Aslund C, Nordquist N, Leppert J, Oreland L. MAOA genotype, family relations and sexual abuse in relation to adolescent alcohol consumption. Addiction biology. 2011;16(2):347-55.
